# Association of genetic polymorphisms with chronic obstructive pulmonary disease in the Chinese Han population: a case–control study

**DOI:** 10.1186/1755-8794-5-64

**Published:** 2012-12-26

**Authors:** Yi Guo, Yi Gong, Chunming Pan, Yanrong Qian, Guochao Shi, Qijian Cheng, Qingyun Li, Lei Ren, Qiuling Weng, Yi Chen, Ting Cheng, Liang Fan, Zhihong Jiang, Huanying Wan

**Affiliations:** 1 Department of Pulmonary Medicine, Rui Jin Hospital, School of Medicine, Shanghai Jiao Tong University, 200025, Shanghai, China; 2 Department of Respiratory Medicine, Hua Shan Hospital, Fu Dan University, 200040, Shanghai, China; 3 State Key Laboratory of Medical Genomics, Molecular Medicine Center, Shanghai Rui Jin Hospital, 200025, Shanghai, China; 4 Department of Respiratory Medicine, Shanghai Jing-an geriatric Hospital, 200042, Shanghai, China; 5 Department of Respiratory Medicine, Shanghai Gong Hui Hospital, 200041, Shanghai, China

**Keywords:** COPD, Single-nucleotide polymorphisms, Genotype, Allele frequencies

## Abstract

**Background:**

Chronic obstructive pulmonary disease (COPD) is influenced by both environmental and genetic factors. Few gene studies of the Chinese population have focused on COPD. We investigated candidate genes associated with susceptibility to COPD in the Chinese Han population.

**Methods:**

A total of 331 COPD patients and 213 control subjects were recruited for this study. Nighty-seven single-nucleotide polymorphisms (SNPs) of 46 genes were selected for genotyping. Genotypes were determined using multiplex polymerase chain reaction (PCR).

**Results:**

Significant differences between patients and healthy controls were observed in the allele frequencies of seven SNPs: rs1205 C, rs2353397 C, rs20541 T, rs2070600 G, rs10947233 G, rs1800629 G, and rs2241712 A. After Bonferroni correction, rs2353397 C was most strongly associated with susceptibility to COPD. Haplotype analysis showed that the frequencies of the GC, GT haplotypes of rs2241718 (*TGF-β1* gene), and rs6957 (*CDC97* gene) were significantly higher in the control group than in the COPD case group (p=1.88×10^-9^); the frequencies of the TT haplotype of rs1205 and rs2808630 (*CRP* gene) were significantly higher in the control group (p=0.0377).

**Conclusion:**

Our study suggests some genetic variants associated with the susceptibility of COPD in the Chinese Han population.

## Background

Chronic obstructive pulmonary disease (COPD) is characterized by progressive airflow limitation driven by an abnormal inflammatory response of the airways to inhaled particles and fumes [1]. The disease is predicted to become the third most common cause of death and the fifth most common cause of disability in the world by 2020 [2]. This disease remains under-recognized and under-diagnosed; the pathogenesis needs to be investigated.

Cigarette smoking is one of the most important risk factors for COPD, but the severity of the disease varies considerably, irrespective of the number of pack-years of smoking. Furthermore, only a minority of smokers (20%) develop the disease clinically, suggesting that in addition to smoking, COPD is partially genetically determined [3,4]. COPD may be caused by a combination of genes and environmental influences. Genes have been associated with COPD in a family-based study, and some previous studies have demonstrated familial aggregation of COPD. The heritability of COPD is estimated to be 40–77% [5]. Some other twin studies have also indicated a genetic contribution to clinically relevant parameters of pulmonary function, such as forced expiratory volume in 1s (FEV1) and forced vital capacity (FVC) [6,7].

Many studies of candidate genes for COPD and pulmonary function have been conducted over the past few years. Above all, genome-wide association studies (GWASs) have identified some loci associated with susceptibility to COPD [8-12] but with varying degrees of reproducibility. Conflicting results among these studies may be attributable to ethnic differences and sample sizes. In the past, candidate gene studies have focused on a single gene or on a few genes in combination; these genes were identified based on prior knowledge or suspected mechanisms of disease pathogenesis. Nonetheless, elucidating the genetics of respiratory disorders is severely hampered by genetic heterogeneity, the low penetrance of individual disease alleles, and the potential for gene–gene and gene–environment interactions. To date, the only proven genetic risk factor for COPD is the severe deficiency of alpha-1-antitrypsin (AAT), which is associated with a predisposition to early onset panacinar (panlobular) emphysema [13].

Furthermore, few gene studies performed on the Chinese population have focused on COPD. However, in China, the disease is increasingly prevalent. A 2007 survey of 20,245 participants in seven regions of China reported the occurrence of COPD in adults aged ≥40 years to be 8.2% [14]. Therefore, more gene-association studies are needed to identify genetic polymorphisms associated with the development of COPD in the Chinese population.

The aim of our current case–control study was to locate genes related to susceptibility to COPD in the Chinese Han population. We aimed to identify loci associated with COPD among 97 single-nucleotide polymorphisms (SNPs) of 46 genes (Table 1).

**Table 1:** Gene location and alleles of 97 single-nucleotide polymorphisms (SNPs)

**SNP_ID**	**Gene**	**Chrom-osome**	**Alleles**	**SNP_ID**	**Gene**	**Chrom-osome**	**Alleles**
rs1800610 [1]	TNF-α	6	C/T	rs673400 [14]	SERPINA2	2	C/G
rs1799964 [1]	TNF-α	6	C/T	rs7583463 [15]	SERPINA2	2	A/C
rs361525 [2]	TNF-α	6	A/G	rs2736100 [8]	TERT	5	G/T
rs1800629 [3]	TNF-α	6	A/G	rs10069690 [8]	TERT	5	C/T
rs2808630 [4]	CRP	1	C/T	rs34829399 [8]	TERT	5	C/T
rs1205 [5]	CRP	1	C/T	rs4246742 [8]	TERT	5	A/T
rs1130864 [4]	CRP	1	C/T	rs2736118 [8]	TERT	5	A/G
rs1059823 [6]	SLC11A1	2	A/G	rs2736122 [8]	TERT	5	C/T
rs1130866 [7]	SFTPB	2	C/T	rs2853677 [8]	TERT	5	C/T
rs2353397 [8]	HHIP	4	C/T	rs2853676 [8]	TERT	5	A/G
rs13147758 [8]	HHIP	4	A/G	rs1881457 [16]	IL-13	5	A/C
rs2035901 [8]	HHIP	4	A/G	rs1295685 [16]	IL-13	5	C/T
rs6537302 [8]	HHIP	4	A/T	rs1800925 [16]	IL-13	5	C/T
rs1032295 [8]	HHIP	4	T/G	rs2066960 [16]	IL-13	5	A/C
rs12504628 [8]	HHIP	4	C/T	rs20541 [16]	IL-13	5	C/T
rs17019336 [8]	HHIP	4	A/T	rs16909898 [8]	PTCH1	9	A/G
rs3749893 [8]	TSPYL-4	6	A/G	rs10512249 [8]	PTCH1	9	C/T
rs4987835 [9]	Bcl-2	18	A/G	rs35621 [17]	ABCC1	16	C/T
rs2292566 [10]	EPHX1	1	A/G	rs2241718 [18]	TGF-β1	19	C/T
rs1051740 [11]	EPHX1	1	C/T	rs56155294 [18]	TGF-β1	19	C/T
rs868966 [11]	EPHX1	1	A/G	rs1800469 [18]	TGF-β1	19	C/T
rs25882 [12]	CSF2	5	C/T	rs2241712 [18]	TGF-β1	19	A/G
rs829259 [13]	PDE4D	5	A/T	rs2277027 [8]	ADAM19	5	A/C
rs6712954 [14]	SERPINA2	2	A/G	rs2280090 [19]	ADAM33	20	A/G
rs2280091 [19]	ADAM33	20	A/G	rs4073 [12]	IL-8	4	A/T
rs1435867 [8]	PID1	2	C/T	rs8192288 [30]	SOD3	4	G/T
rs10498230 [8]	PID1	2	C/T	rs2571445 [20]	TNS1	2	C/T
rs3995090 [20]	HTR4	5	A/C	rs1003349 [31]	MMP14	14	G/T
rs6889822 [8]	HTR4	5	A/G	rs737693 [32]	MMP12	11	A/T
rs1531697 [9]	Bcl-2	18	A/T	rs2276109 [32]	MMP12	11	A/G
rs1042713 [21]	ARDB2	5	A/G	rs1052443 [8]	NT5DC1	6	A/C
rs3024791 [22]	SFTPB	2	A/G	rs10947233 [8]	PPT2	6	G/T
rs511898 [23]	ADAM33	20	C/T	rs1051730 [33]	CHRNA3	15	C/T
rs2853209 [23]	ADAM33	20	A/T	rs11106030 [20]	DCN	12	A/C
rs6555465 [8]	ADCY2	5	A/G	rs584367 [34]	sPLA2s	1	C/T
rs10075508 [13]	PDE4D	5	C/T	rs9904270 [26]	CDC6	17	C/T
rs12899618 [20]	THSD4	15	A/G	rs2395730 [8]	DAAM2	6	A/C
rs3091244 [8]	SFXN1	5	A/C/T	rs3817928 [8]	GPR126	6	A/G
rs8004738 [24]	SERPINA1	14	A/G	rs11155242 [8]	GRP126	6	A/C
rs709932 [24]	SERPINA1	14	A/G	rs7776375 [8]	GPR126	6	A/G
rs4934 [25]	SERPINA3	14	A/G	rs6937121 [8]	GPR126	6	G/T
rs13706 [26]	CDC6	17	A/G	rs1042714 [35]	ARDB2	5	C/G
rs7217852 [26]	CDC6	17	A/G	rs1800796 [36]	IL-6	7	C/G
rs2077464 [26]	CDC6	17	A/G	rs2236307 [31]	MMP14	14	C/T
rs2070600 [20]	AGER	6	A/G	rs2236302 [31]	MMP14	14	C/G
rs6957 [27]	CDC97	19	A/G	rs2230054 [37]	IL-8RB	2	C/T
rs1042522 [28]	P53	17	C/G	rs1422795 [8]	ADAM19	5	A/G
rs1695 [29]	GSTP1	11	A/G	rs6830970 [8]	FAM13A	4	A/G
rs2869967 [8]	FAM13A	4	C/T				

References listed in supplemental document.

## Methods

### Subjects

In total, 331 COPD patients were recruited: 256 from the Department of Respiratory Diseases of Shanghai Rui Jin Hospital, 60 from the Shanghai Jing-an Geriatric Hospital, and 15 from the Shanghai Gong Hui Hospital. COPD was diagnosed according to the criteria established by the NHLBI/WHO Global Initiative for COPD (GOLD) [15]. The diagnoses were based on certain patient parameters (e.g., age≥40 years and smoking history of ≥20 pack-years) and on the presence of relentless and progressive symptoms: cough, productive sputum, and breathlessness over many years; airflow limitation as indicated by FEV1/ FVC≤70%; FEV1 reversibility after the inhalation of salbutamol <12% of the pre-bronchodilator FEV1 (MS-Body Diffusion, Germany). Patients were excluded if they had a comorbid diagnosis such as asthma or lung cancer, or had radiographic abnormalities suggestive of other significant respiratory diseases and any hereditary diseases.

Control subjects (n = 213) were selected from a pool of healthy people who visited the general health checkup center of Shanghai Rui Jin Hospital during the same period. The enrollment criteria for the controls were as follows: age≥40 years, smoker, no known disease, no history of any disease. Lung function was measured at baseline following the American Thoracic Society/European Respiratory Society standard procedure to confirm no evidence of airflow obstruction. All of the COPD patients and control subjects were ethnic Han Chinese. The study protocol was approved by the medical ethics committee of Shanghai Rui Jin Hospital, Shanghai Jiaotong University School of Medicine, and all participants gave written informed consent.

### DNA extraction and genotyping

We chose 97 candidate SNPs identified in previously published GWASs and by searching the dbSNP database of NCBI (references in Additional file 1: Table S1). Their minor allele frequencies (MAFs) were >0.05 in the Han Chinese population we studied.

A 4-ml peripheral blood sample was obtained from each participant for DNA analysis. Plasma was separated by centrifuge and stored at −80°C until further use. We extracted genomic DNA using a QuickGene DNA whole blood kit, (Life Sciences, FUJIFILM, Japan). Any sample with DNA concentration <10 ng/ul was excluded, and another sample was acquired.

For genotyping, we first performed multiplex PCR, a variant of PCR that enables simultaneous amplification of many targets of interest in one reaction by using more than one pair of primers [16]. Mass-ARRAY™ Assay Design 2.0 software was used to design multiplex primers for each SNP: 1st PCR primer, 2nd PCR primer, and UEP primer. The primers of 97 SNPs are shown in Additional file 1: Table S1. Genotyping was achieved using the Mass-Array™ Technology platform of Sequenom, Inc. (San Diego, CA, USA). For quality control, two independent readers interpreted the results, and a random selection of 10% of all samples was retested. No discrepancies were discovered in the replicate tests. All genotyping analyses were blinded with respect to the case/control status, and all samples were analyzed in the same lab and under the same conditions. The results were 100% concordant. Several SNP samples were finally excluded because ≥10% of the genotyping data were missing.

### Data analysis

Data analyses were performed using the Statistical Package for the Social Science 20.0 (SPSS, Inc., Chicago, IL, USA). Continuous variables (age, smoking history, and pulmonary function) were calculated as means (± standard deviation). The two-sided Student’s t-test was used to determine significant differences in clinical data between the COPD cases and the control subjects. The significance level for t tests of clinical information was 0.05. The χ^2^ test and unconditional logistic method were applied to compare genotype and allele frequencies between the two groups, logistic analysis was adjusted for age, gender and smoking. Frequencies were compared, respectively, using a p cutoff of 0.05 (like previous studies) and the Bonferroni correction method for multiple testing. The relative risk associated was estimated as an odds ratio (OR) with a 95% confidence interval (95% CI). Each of the SNPs in the control group was analyzed for Hardy-Weinberg equilibrium (HWE) using chi-square test and exact test, SNPs were excluded from the analysis if they were out of HWE (p≤0.05). Haplotype frequencies and linkage disequilibrium (LD) analyses were evaluated using PHASE and Haploview software.

## Results

### Study population characteristics

The study population characteristics are described in Table 2. They did not significantly differ in sex, age, or smoking history. FEV1 predictive (FEV1%) and FEV1/FVC of the case group were significantly decreased compared with the control group (p<0.05).

**Table 2:** Demographics of COPD patients and control subjects

	**COPD**	**Controls**	**P value**
**Number**	331	213	
**Age**	61±10	58±12	
**Male(%)**	298(90%)	209(98%)	
**Female(%)**	33(10%)	4(2%)	
**Pack-years(±SD)**	41±34	38±17	
**FEV1/FVC**	54±13.8^*^	85±7.6^*^	p<0.05
**FEV1/predicted(%)**	49±18.1^#^	88±17.0^#^	p<0.05

Data were presented as the means ± SD. FEV1, forced expiratory volume in 1 sec; FVC, forced vital capacity; ^*^p<0.05 significant difference vs control.

^#^p<0.05 significant difference vs control.

### Association analysis of each genotype

Eight SNPs (rs361525, rs1042713, rs34829399, rs2853677, rs2571445, rs8192288, rs2066960, and rs2230054) that deviated from HWE in the controls were removed from the association analysis. Thirteen SNPs (rs1130866, rs56155294, rs10498230, rs2035901, rs3091244, rs511898, rs2869967, rs7583463, rs2276109, rs737693, rs9904270, rs4934, and rs6830970) were also eliminated from the analysis due to lack of genotyping data in ≥10% of the sample. Finally, 76 of the 97 SNPs were included in the association analysis. The allele frequencies (Table 3) and the genotype distributions for these SNPs were analyzed in samples from 331 COPD patients and 213 control subjects. Seven SNPs tended to be associated with COPD: rs2353397, rs1800629, rs2241712, rs1205, rs20541, rs2070600, and rs10947233. Among these seven SNPs, after Bonferroni correction, rs2353397 was most strongly associated with susceptibility to COPD. The C allele (rs2353397) of the human hedgehog interacting protein (*HHIP*) gene occurred more frequently in COPD patients (58%) than in the control subjects (29%) (OR = 2.16, 95% CI 1.66–2.81, p<0.0001, p(Bonferroni) <0.0001). The G allele (rs1800629) of the *TNF-α* gene was more frequently detected in COPD patients (95%) versus control subjects (90%) (OR=1.97, 95% CI 1.21–3.21, p=0.0060, p(Bonferroni)=0.4560). The frequency of the A allele (rs2241712) of the *TGF-β1* gene was significantly higher in COPD patients (52%) than in healthy controls (45%) (OR=1.24, 95% CI 0.96–1.59, p=0.0460, p(Bonferroni)=3.7848). The C allele (rs1205) of the *CRP* gene occurred more frequently in COPD patients (47%) compared with control subjects (40%) (OR=1.48, 95% CI 1.14–1.91, p=0.0030, p(Bonferroni)=0.2280). More COPD patients (35%) carried the T allele (rs20541) of the *IL-13* gene than control subjects (28%) (OR=1.36, 95% CI 1.04–1.80, p=0.0280, p(Bonferroni)=2.1280). The G allele (rs2070600) of the *AGER* gene was found more frequently in COPD patients (81%) than in healthy controls (73%) (OR=1.47, 95% CI 1.08–1.98, p=0.0130, p(Bonferroni)=0.9880). The G allele (rs10947233) of the *PPT2* gene occurred more frequently in COPD patients (79%) than in control subjects (72%) (OR=1.51, 95% CI 1.12–2.03, p=0.0060, p(Bonferroni)=0.4560).

**Table 3:** Allele frequencies in COPD and control subjects for SNPs

***SNP***	***Allele***	***Control (n,%)***	***Case (n,%)***	***χ***^***2***^	***P value***	***OR***	***OR(95%CI)***	***P***_***(Bonferroni)***_	***Adjusted P value***	***Adjusted OR***	***Adjusted OR(95%CI)***	***Adjusted P***_***(Bonferroni)***_
**rs1059823**	**G**	139(33)	222(34)	0.0181	0.8929	1.01	0.79-1.32	67.8604	0.8290	0.97	0.74-1.27	63.0040
	**A**	283(67)	440(66)									
**rs1205**	**C**	168(40)	308(47)	5.2168	**0.0223**	1.34	1.04-1.71	1.6948	**0.0030**	1.48	1.14-1.91	0.2280
	**T**	252(60)	346(53)									
**rs17019336**	**A**	136(32)	242(37)	2.1770	0.1401	1.21	0.94-1.57	10.6476	0.0670	1.28	0.98-1.68	5.0920
	**T**	284(68)	416(63)									
**rs1799964**	**T**	333(79)	519(79)	0.0008	0.9772	1.00	0.74-1.36	74.2672	0.8140	0.96	0.71-1.31	61.8640
	**C**	87(21)	135(21)									
**rs1800610**	**T**	71(17)	112(17)	0.0117	0.9137	1.02	0.74-1.41	69.4412	0.9007	0.98	0.70-1.38	68.4532
	**C**	355(83)	550(83)									
**rs2077464**	**T**	271(65)	420(66)	0.1909	0.6621	1.06	0.82-1.37	50.3196	0.8230	1.03	0.79-1.35	62.5480
	**C**	149(35)	218(34)									
**rs2236302**	**C**	369(88)	584(89)	0.2011	0.6539	1.09	0.75-1.59	49.6964	0.4140	1.18	0.80-1.75	31.4640
	**G**	51(12)	74(11)									
**rs2292566**	**A**	125(30)	209(32)	0.4800	0.4884	1.10	0.84-1.43	37.1184	0.7630	1.04	0.79-1.38	57.9880
	**G**	295(70)	449(68)									
**rs2353397**	**C**	123(29)	382(58)	83.3798	**6.8×10**^**-20**^	3.29	2.54-4.28	**5.2×10**^**-18**^	**<0.0001**	2.16	1.66-2.81	**<0.0001**
	**T**	297(71)	280(42)									
**rs25882**	**T**	147(35)	240(36)	0.2421	0.6227	1.07	0.83-1.38	47.3252	0.4650	1.10	0.85-1.44	35.3400
	**C**	273(65)	418(64)									
**rs2808630**	**C**	66(16)	119(18)	1.0136	0.3140	1.18	0.85-1.65	23.8640	0.2120	0.86	0.69-1.09	16.1120
	**T**	354(84)	539(82)									
**rs3749893**	**A**	286(67)	454(69)	0.6232	0.4299	1.11	0.86-1.44	32.6724	0.4510	1.11	0.84-1.46	34.2760
	**G**	140(33)	200(31)									
**rs4987835**	**A**	236(56)	382(60)	1.7185	0.1899	1.19	0.92-1.51	14.4324	0.2950	1.15	0.88-1.49	22.4200
	**G**	184(44)	252(40)									
**rs709932**	**A**	73(17)	131(20)	1.2714	0.2595	1.20	0.87-1.65	19.7220	0.2860	1.19	0.86-1.65	21.7360
	**G**	347(83)	519(80)									
**rs7217852**	**A**	273(65)	434(66)	0.0652	0.7985	1.03	0.80-1.34	60.6860	0.8460	1.03	0.79-1.34	64.2960
	**G**	147(35)	226(34)									
**rs7776375**	**A**	270(63)	438(66)	0.8832	0.3473	1.13	0.88-1.46	26.3948	0.2570	1.17	0.89-1.52	19.5320
	**G**	156(37)	224(34)									
**rs10069690**	**C**	331(80)	520(81)	0.0264	0.8709	1.03	0.75-1.40	66.1884	0.6480	1.08	0.78-1.48	49.2480
	**T**	81(20)	124(19)									
**rs1051740**	**T**	247(60)	403(61)	0.0424	0.8369	1.03	0.79-1.32	63.6044	0.8910	1.02	0.79-1.32	67.7160
	**C**	163(40)	259(39)									
**rs11155242**	**A**	372(90)	604(91)	0.5784	0.4469	1.18	0.77-1.79	33.9644	0.2560	1.28	0.83-1.94	19.4560
	**C**	42(10)	58(9)									
**rs1295685**	**T**	118(29)	221(33)	2.8124	0.0935	1.26	0.96-1.64	7.1060	0.1730	1.21	0.92-1.60	13.1480
	**C**	296(71)	441(67)									
**rs1435867**	**C**	55(13)	90(14)	0.0200	0.8877	1.03	0.72-1.47	67.4652	0.5300	0.89	0.62-1.29	40.5080
	**T**	355(87)	566(86)									
**rs16909898**	**G**	33(8)	54(8)	0.0101	0.9200	1.02	0.65-1.61	69.9200	0.3140	0.79	0.50-1.25	23.8640
	**A**	379(92)	606(92)									
**rs1881457**	**A**	308(74)	495(75)	0.0726	0.7876	1.04	0.78-1.38	59.8576	0.9120	1.02	0.76-1.36	69.3120
	**C**	108(26)	167(25)									
**rs2241718**	**T**	114(28)	206(31)	1.4433	0.2296	1.18	0.90-1.55	17.4496	0.2930	1.16	0.88-1.53	22.2680
	**C**	298(72)	456(69)									
**rs2277027**	**C**	64(15)	106(16)	0.0586	0.8088	1.04	0.74-1.46	61.4688	0.8350	0.96	0.68-1.36	63.4600
	**A**	350(85)	556(84)									
**rs2736100**	**T**	231(57)	368(58)	0.1539	0.6948	1.05	0.82-1.35	52.8048	0.6340	1.06	0.82-1.38	48.1840
	**G**	173(43)	262(42)									
**rs35621**	**C**	305(74)	499(75)	0.1317	0.7167	1.05	0.79-1.40	54.4692	0.3480	1.15	0.86-1.54	26.4480
	**T**	105(26)	163(25)									
**rs3995090**	**C**	288(70)	461(71)	0.1521	0.6965	1.06	0.80-1.39	52.9340	0.4200	1.12	0.84-1.48	31.9200
	**A**	122(30)	185(29)									
**rs4246742**	**A**	244(60)	429(65)	3.0339	0.0815	1.25	0.97-1.61	6.1940	0.0510	1.32	1.01-1.71	3.8760
	**T**	166(40)	233(35)									
**rs6712954**	**G**	321(78)	545(82)	3.1679	0.0751	1.32	0.97-1.79	5.7076	0.0560	1.38	1.01-1.89	4.2560
	**A**	91(22)	117(18)									
**rs829259**	**A**	137(33)	233(35)	0.4250	0.5145	1.09	0.84-1.41	39.1020	0.9300	1.01	0.77-1.32	70.6800
	**T**	275(67)	429(65)									
**rs10075508**	**T**	69(16)	108(17)	0.0253	0.8736	1.03	0.74-1.43	66.3936	0.9070	1.02	0.72-1.44	68.9320
	**C**	357(84)	544(83)									
**rs10512249**	**T**	33(8)	52(8)	0.0606	0.8056	1.06	0.67-1.67	61.2256	0.4950	1.16	0.75-1.80	37.6200
	**C**	383(92)	570(92)									
**rs12899618**	**G**	370(89)	579(89)	0.0806	0.7765	1.06	0.72-1.56	59.0140	0.6010	1.11	0.75-1.65	45.6760
	**A**	48(11)	71(11)									
**rs13706**	**G**	272(65)	427(65)	0.0598	0.8068	1.03	0.80-1.34	61.3168	0.8300	0.97	0.75-1.27	63.0800
	**A**	148(35)	225(35)									
**rs1531697**	**A**	255(61)	411(63)	0.5370	0.4637	1.10	0.85-1.41	35.2412	0.4750	1.10	0.85-1.43	36.1000
	**T**	163(39)	239(37)									
**rs1800925**	**T**	62(15)	105(17)	0.8620	0.3531	1.18	0.84-1.66	26.8356	0.1000	1.32	0.94-1.85	7.6000
	**C**	352(85)	507(83)									
**rs3024791**	**G**	388(93)	616(95)	1.5299	0.2161	1.39	0.82-2.34	16.4236	0.3820	1.25	0.76-2.06	29.0320
	**A**	28(7)	32(5)									
**rs6537302**	**A**	310(75)	480(77)	0.7206	0.3959	1.13	0.85-1.51	30.0884	0.9110	1.10	0.76-1.36	69.2360
	**T**	104(25)	142(23)									
**rs6555465**	**G**	195(46)	310(48)	0.4399	0.5072	1.09	0.85-1.39	38.5472	0.5010	1.09	0.85-1.41	38.0760
	**A**	231(54)	338(52)									
**rs673400**	**C**	178(43)	278(43)	0.0062	0.9371	1.01	0.79-1.30	71.2196	0.9280	0.99	0.76-1.28	70.5280
	**G**	238(57)	368(57)									
**rs6889822**	**G**	268(64)	417(65)	0.0594	0.8073	1.03	0.80-1.34	61.3548	0.5000	1.10	0.84-1.43	38.0000
	**A**	148(36)	223(35)									
**rs8004738**	**G**	184(44)	275(44)	0.0092	0.9236	1.01	0.79-1.30	70.1936	0.6650	1.01	0.82-1.37	50.5400
	**A**	232(56)	351(56)									
**rs1003349**	**G**	238(57)	392(60)	0.8897	0.3456	1.13	0.88-1.45	26.2656	0.2340	1.17	0.90-1.51	17.7840
	**T**	178(43)	260(40)									
**rs1032295**	**T**	320(75)	523(80)	3.1870	0.0742	1.30	0.97-1.74	5.6392	0.1130	1.28	0.94-1.73	8.5880
	**G**	106(25)	133(20)									
**rs1042522**	**C**	184(44)	304(47)	0.8170	0.3660	1.12	0.88-1.43	27.8160	0.4090	1.11	0.86-1.44	31.0840
	**G**	236(56)	348(53)									
**rs1052443**	**C**	281(67)	457(71)	1.7602	0.1846	1.20	0.92-1.56	14.0296	0.1610	1.22	0.93-1.60	12.2360
	**A**	139(33)	189(29)									
**rs12504628**	**T**	305(72)	475(72)	0.0847	0.7710	1.04	0.79-1.37	58.5960	0.9810	1.04	0.76-1.33	74.5560
	**C**	121(28)	181(28)									
**rs1695**	**G**	72(17)	126(19)	0.6673	0.4140	1.14	0.83-1.57	31.4640	0.4650	1.13	0.82-1.57	35.3400
	**A**	346(83)	530(81)									
**rs1800469**	**C**	182(44)	315(48)	2.1252	0.1449	1.20	0.94-1.54	11.0124	0.2010	1.74	1.35-2.27	15.2760
	**T**	234(56)	337(52)									
**rs20541**	**T**	118(28)	228(35)	5.3633	**0.0206**	1.37	1.05-1.79	1.5656	**0.0280**	1.36	1.04-1.80	2.1280
	**C**	302(72)	426(65)									
**rs2070600**	**G**	312(73)	529(81)	8.1712	**0.0043**	1.52	1.14-2.03	0.3268	**0.0130**	1.47	1.08-1.98	0.9880
	**A**	114(27)	127(19)									
**rs2853209**	**A**	191(45)	305(47)	0.1953	0.6586	1.06	0.83-1.35	50.0536	0.9890	0.10	0.77-1.29	75.1640
	**T**	231(55)	349(53)									
**rs4073**	**A**	185(44)	300(46)	0.5198	0.4709	1.10	0.86-1.40	35.7884	0.2530	1.16	0.90-1.50	19.2280
	**T**	235(56)	348(54)									
**rs6937121**	**T**	254(60)	423(65)	2.1263	0.1448	1.21	0.94-1.56	11.0048	0.1720	1.20	0.92-1.56	13.0720
	**G**	166(40)	229(35)									
**rs6957**	**G**	150(36)	241(37)	0.0802	0.7771	1.04	0.80-1.34	59.0596	0.6830	1.06	0.81-1.38	51.9080
	**A**	268(64)	415(63)									
**rs1051730**	**C**	403(97)	641(97)	0.2343	0.6284	1.20	0.57-2.52	47.3252	0.6480	1.17	0.60-2.29	49.2480
	**T**	11(3)	21(3)									
**rs10947233**	**G**	299(72)	526(79)	7.4524	**0.0063**	1.49	1.12-1.98	0.4788	**0.0060**	1.51	1.12-2.03	0.4560
	**T**	115(28)	136(21)									
**rs11106030**	**C**	355(85)	560(85)	0.0013	0.9716	1.01	0.71-1.42	73.8416	0.7030	1.07	0.75-1.52	53.4280
	**A**	63(15)	100(15)									
**rs1130864**	**T**	23(6)	43(7)	0.6081	0.4355	1.23	0.73-2.07	33.0980	0.3890	1.24	0.77-2.00	29.5640
	**C**	389(94)	591(93)									
**rs1800629**	**G**	379(90)	627(95)	7.8793	**0.0050**	1.94	1.21-3.10	0.3800	**0.0060**	1.97	1.21-3.21	0.4560
	**A**	41(10)	35(5)									
**rs2241712**	**A**	188(45)	342(52)	3.9820	**0.0460**	1.28	1.00-1.64	3.4960	**0.0498**	1.24	0.96-1.59	3.7848
	**G**	226(55)	320(48)									
**rs2280090**	**G**	395(94)	629(95)	0.9844	0.3211	1.30	0.77-2.20	24.4036	0.4640	1.22	0.72-2.06	35.2640
	**A**	27(6)	33(5)									
**rs2395730**	**A**	119(28)	209(32)	1.3886	0.2386	1.17	0.90-1.54	18.1336	0.0850	1.28	0.97-1.69	6.4600
	**C**	303(72)	453(68)									
**rs2736118**	**A**	397(94)	630(95)	0.6150	0.4329	1.24	0.72-2.12	32.9004	0.2850	1.36	0.78-2.37	21.6600
	**G**	25(6)	32(5)									
**rs2736122**	**C**	388(94)	632(95)	1.5783	0.2090	1.41	0.82-2.42	15.884	0.0510	1.77	1.02-3.07	3.8760
	**T**	26(6)	30(5)									
**rs3817928**	**A**	370(89)	596(90)	0.1202	0.7288	1.07	0.72-1.61	55.3888	0.4410	1.17	0.78-1.76	33.5160
	**G**	44(11)	66(10)									
**rs584367**	**T**	91(22)	152(23)	0.1399	0.7083	1.06	0.79-1.42	53.8308	0.8590	1.03	0.76-1.39	65.2840
	**C**	323(78)	510(77)									
**rs1042714**	**C**	374(90)	607(92)	1.1947	0.2744	1.27	0.83-1.96	20.8544	0.1440	1.39	0.90-2.14	10.9440
	**G**	40(10)	374(90)									
**rs13147758**	**A**	283(69)	464(71)	0.8780	0.3487	1.14	0.87-1.49	26.5012	0.3840	1.13	0.86-1.49	29.1840
	**G**	129(31)	186(29)									
**rs1422795**	**G**	61(15)	108(16)	0.5395	0.4626	1.14	0.81-1.60	35.1576	0.8690	1.03	0.73-1.46	66.0440
	**A**	353(85)	550(84)									
**rs1800796**	**C**	293(71)	473(72)	0.1539	0.6948	1.06	0.80-1.39	52.8048	0.8250	1.03	0.78-1.36	62.7000
	**G**	121(29)	185(28)									
**rs2236307**	**C**	169(41)	286(43)	0.7270	0.3938	1.11	0.87-1.43	29.9288	0.4150	1.11	0.86-1.44	31.5400
	**T**	245(59)	372(57)									
**rs2280091**	**A**	383(93)	611(93)	0.1518	0.6968	1.10	0.68-1.77	52.9568	0.5020	1.17	0.74-1.87	38.1520
	**G**	31(7)	45(7)									
**rs2853676**	**G**	335(81)	544(83)	0.4538	0.5005	1.12	0.81-1.54	38.0380	0.2770	1.20	0.86-1.67	21.0520
	**A**	77(19)	112(17)									
**rs868966**	**A**	205(50)	337(51)	0.2934	0.5880	1.07	0.84-1.37	44.6880	0.7890	1.04	0.80-1.34	59.9640
	**G**	209(50)	321(49)									

Chi-square test and logistic analysis were used.

Logistic analysis was adjusted by potential confounders including age, gender, smoking history.

The p value with bold letters indicate those allele frequencies with significant differences between COPD and controls.

For analysis of genotypic association of these seven SNPs under certain genotype models (Table 4), rs2353397 TT protected subjects from the disease; CC, CT carriers were more susceptible to COPD (OR=1.01, 95% CI 0.79–1.32, p<0.0001). The rs1800629 GG homozygous carriers exhibited an increased susceptibility to the disease compared with AA, GA carriers (OR=1.90, 95% CI 1.12–3.21, p=0.0170). The rs1205 CC, CT genotype increased risk for COPD compared with the TT homozygous genotype (OR=1.82, 95% CI 1.21–2.73, p=0.0040). Individuals carrying the rs20541 TT, CT genotype were at a significantly higher risk for COPD than were healthy subjects carrying the CC genotype (OR=1.47, 95% CI 1.01–2.13, p=0.0450). The rs2070600 GG homozygous carriers tended to develop COPD more frequently that AA, GA carriers (OR=1.55, 95% CI 1.06–2.26, p=0.0240). rs10947233 GG, GT carriers were associated with susceptibility to COPD compared with TT carriers (OR=3.30, 95% CI 1.47–7.44, p=0.0040).GG carriers versus GT, TT carriers (OR=1.56, 95% CI 1.07–2.27, p=0.0200).

**Table 4:** Analysis of Genotypic Association of SNPs identified under Genetic Models

**SNP**	**Genotype model**	**Control (n,%)**	**Case (n,%)**	P value*	OR*	OR(95%CI) *
**rs2353397**	**CC+CT**	104(50)	261(79)	**<0.0001**	1.01	0.79-1.32
	**TT**	106(50)	70(21)			
**rs2070600**	**GG**	100(47)	213(65)	**0.0240**	1.55	1.06-2.26
	**GA+AA**	113(53)	115(35)			
**rs10947233**	**GG+GT**	191(92)	319(96)	**0.0040**	3.30	1.47-7.44
	**TT**	16(8)	12(4)			
**rs10947233**	**GG**	108(52)	207(63)	**0.0200**	1.56	1.07-2.27
	**GT+TT**	99(48)	124(37)			
**rs1800629**	**GG**	171(81)	296(89)	**0.0170**	1.90	1.12-3.21
	**GA+AA**	39(19)	35(11)			
**rs1205**	**CC+CT**	134(64)	238(73)	**0.0040**	1.82	1.21-2.73
	**TT**	76(36)	89(27)			
**rs20541**	**TT+CT**	99(47)	189(58)	**0.0450**	1.47	1.01-2.13
	**CC**	111(53)	138(42)			

* Logistic regression analysis was adjusted by potential confounders including age, gender, smoking history.

The complete genotype distributions of the other SNPs are listed in Table 5. Frequencies under different genotypic models of each SNP were compared between the COPD group and the control group.

**Table 5:** Genotype frequencies of each SNP in COPD and control subjects for SNPs

***SNP***	***Allele***	***Control (n,%)***	***Case (n,%)***	***Adjusted P value***	***Adjusted OR***	***Adjusted OR(95%CI)***	***Adjusted P***_***(Bonferroni)***_
**rs1059823**	**GG**	24(11)	39(11)	0.8120	0.93	0.52-1.67	61.7120
	**GA+AA**	187(89)	292(89)				
	**GG+GA**	115(55)	183(55)	0.8760	0.97	0.68-1.39	66.5760
	**AA**	96(45)	148(45)				
**rs17019336**	**AA**	26(12)	35(11)	0.0100	0.14	0.09-0.22	0.7600
	**TA+TT**	184(88)	294(89)				
	**AA+AT**	110(52)	207(63)	0.0210	1.54	1.07-2.21	1.5960
	**TT**	100(48)	122(37)				
**rs1799964**	**TT**	130(62)	206(63)	0.8010	0.95	0.66-1.38	60.8760
	**TC+CC**	80(38)	121(37)				
	**TT+TC**	203(97)	313(96)	0.9180	0.96	0.41-2.23	69.7680
	**CC**	7(3)	14(4)				
**rs1800610**	**TT**	9(4)	13(4)	0.9940	1.00	0.41-2.44	75.5440
	**TC+CC**	204(96)	318(96)				
	**TT+TC**	62(29)	99(30)	0.7350	1.07	0.72-1.59	55.8600
	**CC**	151(71)	232(70)				
**rs2077464**	**TT**	85(40)	138(43)	0.6620	1.09	0.75-1.57	50.3120
	**TC+CC**	125(60)	181(57)				
	**TT+TC**	186(89)	282(88)	0.8190	0.94	0.53-1.64	62.2440
	**CC**	24(11)	37(12)				
**rs2236302**	**CC+GC**	208(99)	327(99)	0.8210	1.26	0.18-8.99	62.3960
	**GG**	2(1)	2(1)				
	**CC**	161(77)	257(78)	0.4060	1.20	0.78-1.84	30.8560
	**GG+GC**	49(23)	72(22)				
**rs2292566**	**AA**	21(10)	36(11)	0.7410	1.11	0.61-2.0	56.3160
	**GG+AG**	189(90)	293(89)				
	**AA+AG**	106(50)	156(47)	0.8610	1.03	0.72-1.48	65.4360
	**GG**						
**rs2353397**	**CC**	19(9)	121(37)	0.4850	1.24	0.68-2.28	36.8600
	**CT+TT**	191(91)	210(63)				
	**CC+CT**	104(50)	261(79)	0.3740	1.18	0.82-1.69	28.4240
	**TT**	106(50)	70(21)				
**rs25882**	**TT**	30(14)	46(14)	0.9400	1.02	0.61-1.71	71.4400
	**CT+CC**	180(86)	283(86)				
	**TT+CT**	117(56)	194(59)	0.3490	1.19	0.83-1.72	26.5240
	**CC**	93(44)	135(41)				
**rs2808630**	**CC**	4(2)	10(3)	0.4820	0.90	0.66-1.22	36.6320
	**CT+TT**	206(98)	319(97)				
	**CC+CT**	62(30)	109(33)	0.2200	0.80	0.55-1.15	16.7200
	**TT**	148(70)	220(67)				
**rs3749893**	**AA**	100(47)	161(50)	0.4510	1.11	0.85-1.46	34.2760
	**AG+GG**	113(53)	166(50)				
	**AA+AG**	186(87)	293(90)	0.6830	1.08	0.75-1.55	51.9080
	**GG**	27(13)	34(10)				
**rs4987835**	**AA**	65(31)	121(38)	0.2280	1.27	0.86-1.86	17.3280
	**AG+GG**	145(69)	196(62)				
	**AA+AG**	171(81)	261(80)	0.6900	1.10	0.69-1.76	52.4400
	**GG**	39(19)	56(20)				
**rs709932**	**AA**	8(4)	16(5)	0.7950	1.13	0.45-2.84	60.4200
	**AG+GG**	202(96)	309(95)				
	**AG+AA**	65(31)	115(35)	0.2570	1.25	0.85-1.82	19.5320
	**GG**	145(69)	210(65)				
**rs7217852**	**AA**	86(41)	141(43)	0.6180	1.01	0.76-1.58	46.9680
	**AG+GG**	124(59)	189(57)				
	**AA+AG**	187(89)	293(89)	0.7000	0.89	0.51-1.57	53.2000
	**GG**	23(11)	37(11)				
**rs7776375**	**AA**	83(39)	148(45)	0.0900	1.37	0.95-1.98	6.8400
	**AG+GG**	130(61)	183(55)				
	**AA+AG**	187(39)	290(45)	0.7940	0.93	0.54-1.6	60.3440
	**GG**	26(12)	41(12)				
**rs10069690**	**TT**	12(6)	7(2)	0.7100	0.93	0.64-1.35	53.9600
	**CT+CC**	194(94)	315(98)				
	**TT+CT**	69(33)	117(36)	0.0240	3.14	1.17-9.92	1.8240
	**CC**	137(67)	205(64)				
**rs1051740**	**TT**	72(35)	123(37)	0.5730	1.12	0.76-1.63	43.5480
	**TC+CC**	133(65)	208(63)				
	**TT+TC**	175(85)	280(85)	0.6070	0.88	0.53-1.45	46.1320
	**CC**	30(15)	51(15)				
**rs11155242**	**AA**	169(82)	276(83)	0.3060	1.27	0.80-2.01	23.2560
	**AC+CC**	38(18)	55(17)				
	**AA+AC**	203(98)	328(99)	0.5030	1.68	0.37-7.56	38.2280
	**CC**	4(2)	3(1)				
**rs1295685**	**TT**	19(9)	37(12)	0.4470	1.27	0.69-2.32	33.9720
	**TC+CC**	188(91)	294(88)				
	**TT+CT**	99(48)	184(56)	0.1880	1.27	0.89-1.83	14.2880
	**CC**	108(52)	147(44)				
**rs1435867**	**CC**	5(2)	7(2)	0.7660	0.83	0.24-2.85	58.2160
	**CT+TT**	200(98)	321(98)				
	**CC+CT**	50(24)	83(25)	0.5380	0.88	0.58-1.33	40.8880
	**TT**	155(76)	245(75)				
**rs16909898**	**GG**	2(1)	1(0.3)	0.4560	0.40	0.03-4.45	34.6560
	**GA+AA**	204(99)	329(99.7)				
	**GG+GA**	31(15)	53(16)	0.3580	0.80	0.49-1.30	27.2080
	**AA**	175(85)	277(84)				
**rs1881457**	**AA**	117(56)	187(56)	0.9540	0.99	0.69-1.42	72.5040
	**AC+CC**	91(44)	144(44)				
	**AA+AC**	191(92)	308(93)	0.7340	1.13	0.57-2.22	55.7840
	**CC**	17(8)	23(7)				
**rs2241718**	**TT**	10(5)	26(8)	0.1380	1.80	0.83-3.88	10.4880
	**TC+CC**	196(95)	305(92)				
	**TT+TC**	104(50)	180(54)	0.5480	1.12	0.78-1.60	41.6480
	**CC**	102(50)	151(46)				
**rs2277027**	**CC**	5(2)	10(3)	0.4990	1.47	0.49-4.44	37.9240
	**CA+AA**	202(98)	321(97)				
	**CC+CA**	59(29)	96(29)	0.6170	0.90	0.61-1.34	46.8920
	**AA**	148(71)	235(71)				
**rs2736100**	**TT**	72(36)	108(34)	0.7840	0.95	0.65-1.39	59.5840
	**TG+GG**	130(64)	207(66)				
	**TT+TG**	159(79)	260(83)	0.2490	1.32	0.83-2.10	18.9240
	**GG**	43(21)	55(17)				
**rs35621**	**CC**	114(56)	185(56)	0.3820	1.18	0.82-1.69	29.0320
	**CT+TT**	91(44)	146(44)				
	**CC+CT**	191(93)	314(95)	0.5710	1.25	0.58-2.67	43.3960
	**TT**	14(7)	17(5)				
**rs3995090**	**CC**	98(48)	163(50)	0.2110	1.26	0.88-1.80	16.0360
	**CA+AA**	107(52)	160(50)				
	**CC+CA**	190(93)	298(92)	0.6630	0.86	0.43-1.70	50.3880
	**AA**	15(7)	25(8)				
**rs4246742**	**AA**	71(35)	136(41)	0.0550	1.44	0.99-2.10	4.1800
	**AT+TT**	134(65)	195(59)				
	**AA+AT**	173(84)	293(89)	0.1700	1.44	0.86-2.42	12.9200
	**TT**	32(16)	38(11)				
**rs6712954**	**GG**	128(62)	218(66)	0.1940	1.28	0.88-1.86	14.7440
	**GA+AA**	78(38)	113(34)				
	**GG+GA**	193(94)	327(99)	0.0130	4.25	1.37-13.24	0.9880
	**AA**	13(6)	4(1)				
**rs829259**	**AA**	24(12)	35(11)	0.4770	0.81	0.45-1.46	36.2520
	**AT+TT**	182(88)	296(89)				
	**AA+AT**	113(55)	198(60)	0.5920	1.11	0.77-1.59	44.9920
	**TT**	93(45)	133(40)				
**rs10075508**	**TT**	4(2)	6(2)	0.3660	0.49	0.10-2.30	27.8160
	**TC+CC**	209(98)	320(98)				
	**TT+TC**	65(31)	102(31)	0.7280	1.07	0.73-1.58	55.3280
	**CC**	148(69)	224(69)				
**rs10512249**	**TT**	2(1)	1(0.3)	0.4560	0.40	0.04-4.45	34.6560
	**TC+CC**	206(99)	310(99.7)				
	**TT+TC**	31(15)	51(16)	0.4150	1.27	0.71-2.27	31.5400
	**CC**	177(85)	260(84)				
**rs12899618**	**GG**	162(78)	259(79)	0.3010	1.26	0.82-1.94	22.8760
	**GA+AA**	47(32)	66(21)				
	**GG+GA**	208(99)	320(98)	0.1430	0.20	0.02-1.71	10.8680
	**AA**	1(1)	5(2)				
**rs13706**	**GG**	85(40)	138(42)	0.7230	1.07	0.74-1.54	54.9480
	**GA+AA**	125(60)	188(58)				
	**GG+GA**	187(89)	289(89)	0.3210	0.76	0.43-1.31	24.3960
	**AA**	23(11)	37(11)				
**rs1531697**	**AA**	75(36)	134(41)	0.2920	1.22	0.84-1.77	22.1920
	**AT+TT**	134(64)	191(59)				
	**AA+AT**	180(86)	277(85)	0.9290	0.98	0.58-1.645	70.6040
	**TT**	29(14)	48(15)				
**rs1800925**	**TT**	6(3)	12(4)	0.7160	1.22	0.43-3.47	54.4160
	**TC+CC**	201(97)	294(96)				
	**TT+TC**	56(27)	93(31)	0.0790	1.41	0.96-2.08	6.0040
	**CC**	151(73)	213(69)				
**rs3024791**	**GG**	181(87)	293(90)	0.3770	1.27	0.75-2.14	28.6520
	**GA+AA**	27(13)	31(10)				
	**GG+GA**	207(99.5)	323(99.7)	0.8760	1.25	0.08-20.07	66.5760
	**AA**	1(0.5)	1(0.3)				
**rs6537302**	**AA**	115(55)	194(62)	0.5150	1.13	0.79-1.62	39.1400
	**AT+TT**	92(45)	117(38)				
	**AA+AT**	195(94)	286(92)	0.3070	0.68	0.33-1.41	23.3320
	**TT**	12(6)	25(8)				
**rs6555465**	**GG**	40(19)	83(26)	0.1920	1.34	0.86-2.10	14.5920
	**GA+AA**	173(81)	241(74)				
	**GG+GA**	155(73)	227(70)	0.8710	0.97	0.65-1.45	66.1960
	**AA**	58(27)	97(30)				
**rs673400**	**CC**	0(0)	0(0)	NA	NA	NA	NA
	**CG+GG**	208(100)	323(100)				
	**CC+CG**	178(86)	278(86)	0.8190	0.94	0.56-1.57	62.2440
	**GG**	30(14)	45(14)				
**rs6889822**	**GG**	84(40)	133(42)	0.4800	1.14	0.79-1.65	36.4800
	**GA+AA**	124(60)	187(58)				
	**GG+GA**	184(88)	284(89)	0.7550	1.10	0.62-1.95	57.3800
	**AA**	24(12)	36(11)				
**rs8004738**	**GG**	45(22)	68(22)	0.7910	1.06	0.69-1.64	60.1160
	**GA+AA**	163(78)	245(78)				
	**GG+GA**	139(67)	207(66)	0.6880	1.08	0.74-1.60	52.2880
	**AA**	69(33)	106(34)				
**rs1003349**	**GG**	68(33)	122(37)	0.1880	1.29	0.88-1.89	14.2880
	**GT+TT**	140(67)	204(63)				
	**GG+GT**	170(82)	270(83)	0.6130	1.13	0.70-1.83	46.5880
	**TT**	38(18)	56(17)				
**rs1032295**	**TT**	119(56)	206(63)	0.1570	1.30	0.90-1.88	11.9320
	**TG+GG**	94(44)	122(37)				
	**TT+TG**	201(94)	317(97)	0.2380	1.70	0.70-4.12	18.0880
	**GG**	12(6)	11(3)				
**rs1042522**	**CC**	37(18)	60(18)	0.8050	1.06	0.66-1.70	61.1800
	**GC+GG**	173(82)	266(82)				
	**CC+CG**	147(70)	244(75)	0.2860	1.25	0.83-1.86	21.7360
	**GG**	63(30)	82(25)				
**rs1052443**	**CC**	96(46)	163(50)	0.2950	1.21	0.85-1.74	22.4200
	**CA+AA**	114(54)	160(50)				
	**CC+CA**	185(88)	294(91)	0.1970	1.49	0.81-2.71	14.9720
	**AA**	25(12)	29(9)				
**rs12504628**	**TT**	107(50)	166(50)	0.9000	0.98	0.68-1.40	68.4000
	**TC+CC**	106(50)	162(50)				
	**TT+TC**	198(93)	309(94)	0.7810	1.11	0.54-2.27	59.3560
	**CC**	15(7)	19(6)				
**rs1695**	**GG**	5(2)	10(3)	0.4990	1.47	0.48-4.44	37.9240
	**GA+AA**	204(98)	318(97)				
	**GG+GA**	67(32)	116(35)	0.5520	1.12	0.77-1.64	41.9520
	**AA**	142(68)	212(65)				
**rs1800469**	**CC**	39(19)	74(23)	0.9960	1.34	0.98-1.95	75.6960
	**TC+TT**	169(81)	252(77)				
	**CC+TC**	143(69)	241(74)	0.2530	1.26	0.85-1.88	19.2280
	**TT**	65(31)	85(26)				
**rs2853209**	**AA**	42(20)	68(21)	0.4560	1.18	0.76-1.84	34.6560
	**AT+TT**	169(80)	251(79)				
	**AA+AT**	149(71)	232(72)	0.4850	0.87	0.59-1.28	36.8600
	**TT**	62(29)	92(28)				
**rs4073**	**AA**	36(17)	68(21)	0.2460	1.32	0.83-2.09	18.6960
	**AT+TT**	174(83)	256(79)				
	**AA+AT**	149(71)	232(72)	0.3260	1.22	0.82-1.83	24.7760
	**TT**	61(29)	92(28)				
**rs6937121**	**TT**	72(34)	141(43)	0.0280	1.52	1.05-2.20	2.1280
	**TG+GG**	138(66)	185(57)				
	**TT+TG**	182(87)	282(86)	0.6680	0.89	0.53-1.50	50.7680
	**GG**	28(13)	44(14)				
**rs6957**	**GG**	21(10)	40(12)	0.2810	1.37	0.77-2.43	21.3560
	**GA+AA**	188(90)	288(88)				
	**GG+GA**	129(62)	201(61)	0.8730	0.97	0.67-1.41	66.3480
	**AA**	80(38)	127(39)				
**rs1051730**	**CC**	196(95)	311(94)	0.5330	1.25	0.62-2.50	40.5080
	**CT+TT**	11(5)	20(6)				
	**CC+CT**	207(100)	330(99.7)	NA	NA	NA	NA
	**TT**	0(0)	1(0.3)				
**rs11106030**	**CC**	153(73)	237(72)	0.9350	1.02	0.68-1.52	71.0600
	**CA+AA**	56(27)	93(28)				
	**CC+CA**	202(96)	323(98)	0.3350	1.77	0.55-5.66	25.4600
	**AA**	7(4)	7(2)				
**rs1130864**	**TT**	0(0)	0(0)	NA	NA	NA	NA
	**TC+CC**	206(100)	317(100)				
	**TT+TC**	23(11)	43(14)	0.3720	1.26	0.76-2.07	28.2720
	**CC**	183(89)	274(86)				
**rs2241712**	**AA**	44(21)	92(28)	0.1560	1.37	0.89-2.10	11.8560
	**AG+GG**	163(79)	239(72)				
	**AA+AG**	144(69)	250(76)	0.2290	1.28	0.86-1.92	17.4040
	**GG**	63(31)	81(24)				
**rs2280090**	**GG**	185(88)	299(90)	0.4650	1.23	0.71-2.14	35.3400
	**GA+AA**	26(12)	32(10)				
	**GG+GA**	210(99.5)	330(99.4)	0.8760	1.25	0.08-20.07	66.5760
	**AA**	1(0.5)	1(0.6)				
**rs2395730**	**AA**	20(9)	48(15)	0.0490	1.77	1.00-3.13	3.7240
	**AC+CC**	191(91)	283(85)				
	**AA+AC**	99(9)	161(49)	0.3610	1.18	0.82-1.70	27.4360
	**CC**	112(53)	170(51)				
**rs2736118**	**AA**	186(88)	299(90)	0.2740	1.38	0.78-2.45	20.8240
	**AG+GG**	25(12)	32(10)				
	**AA+AG**	211(100)	331(100)	NA	NA	NA	NA
	**GG**	0(0)	0(0)				
**rs2736122**	**CC**	181(87)	301(90)	0.035	1.84	1.04-3.24	2.6600
	**CT+TT**	26(13)	30(10)				
	**CC+CT**	181(100)	331(100)	NA	NA	NA	NA
	**TT**	0(0)	0(0)				
**rs3817928**	**AA**	166(81)	268(81)	0.4490	1.19	0.76-1.85	34.1240
	**AG+GG**	41(19)	63(19)				
	**AA+AG**	204(99)	328(99)	0.7860	1.25	0.25-6.26	59.7360
	**GG**	3(1)	3(1)				
**rs584367**	**TT**	10(5)	14(4)	0.5010	0.73	0.30-1.82	38.0760
	**TC+CC**	197(95)	317(96)				
	**TT+TC**	81(39)	138(42)	0.6410	1.10	0.76-1.57	48.7160
	**CC**	126(61)	193(58)				
**rs1042714**	**CC**	168(81)	280(85)	0.1020	1.48	0.93-2.36	7.7520
	**GC+GG**	39(19)	49(15)				
	**CC+GC**	206(99)	327(99)	0.6990	0.62	0.06-6.90	53.1240
	**GG**	1(1)	2(1)				
**rs13147758**	**AA**	102(50)	168(52)	0.6310	1.09	0.76-1.57	47.9560
	**AG+GG**	104(50)	157(48)				
	**AA+AG**	181(88)	296(91)	0.2920	1.38	0.76-2.49	22.1920
	**GG**	25(12)	29(9)				
**rs1422795**	**GG**	5(2)	11(3)	0.4990	1.47	0.48-4.44	37.9240
	**GA+AA**	202(98)	313(97)				
	**GG+GA**	56(27)	97(29)	0.9400	0.99	0.66-1.46	71.4400
	**AA**	151(73)	232(71)				
**rs1800796**	**CC**	110(53)	168(51)	0.5080	0.89	0.62-1.27	38.6080
	**CG+GG**	97(47)	161(49)				
	**CC+CG**	183(88)	305(97)	0.1060	1.68	0.90-3.16	8.0560
	**GG**	24(12)	24(3)				
**rs2236307**	**CC**	41(20)	62(19)	0.9460	0.98	0.62-1.55	71.8960
	**CT+TT**	166(80)	267(81)				
	**CC+CT**	128(62)	224(68)	0.2150	1.27	0.87-1.86	16.3400
	**TT**	79(38)	105(32)				
**rs2280091**	**AA**	178(86)	283(86)	0.6730	1.11	0.68-1.82	51.1480
	**AG+GG**	29(14)	45(14)				
	**AA+AG**	205(99)	328(100)	NA	NA	NA	NA
	**GG**	2(1)	0(0)				
**rs2853676**	**GG**	137(67)	225(69)	0.3510	1.20	0.82-1.75	26.6760
	**GA+AA**	69(33)	103(31)				
	**GG+GA**	198(97)	319(98)	0.3930	1.58	0.56-4.46	29.8680
	**AA**	8(3)	9(2)				
**rs868966**	**AA**	56(27)	84(26)	0.6620	0.91	0.60-1.38	50.3120
	**AG+GG**	151(73)	245(74)				
	**AA+AG**	149(72)	253(77)	0.3970	1.20	0.79-1.80	30.1720
	**GG**	58(28)	76(23)				

### Haplotype analysis

**Figure 1:**
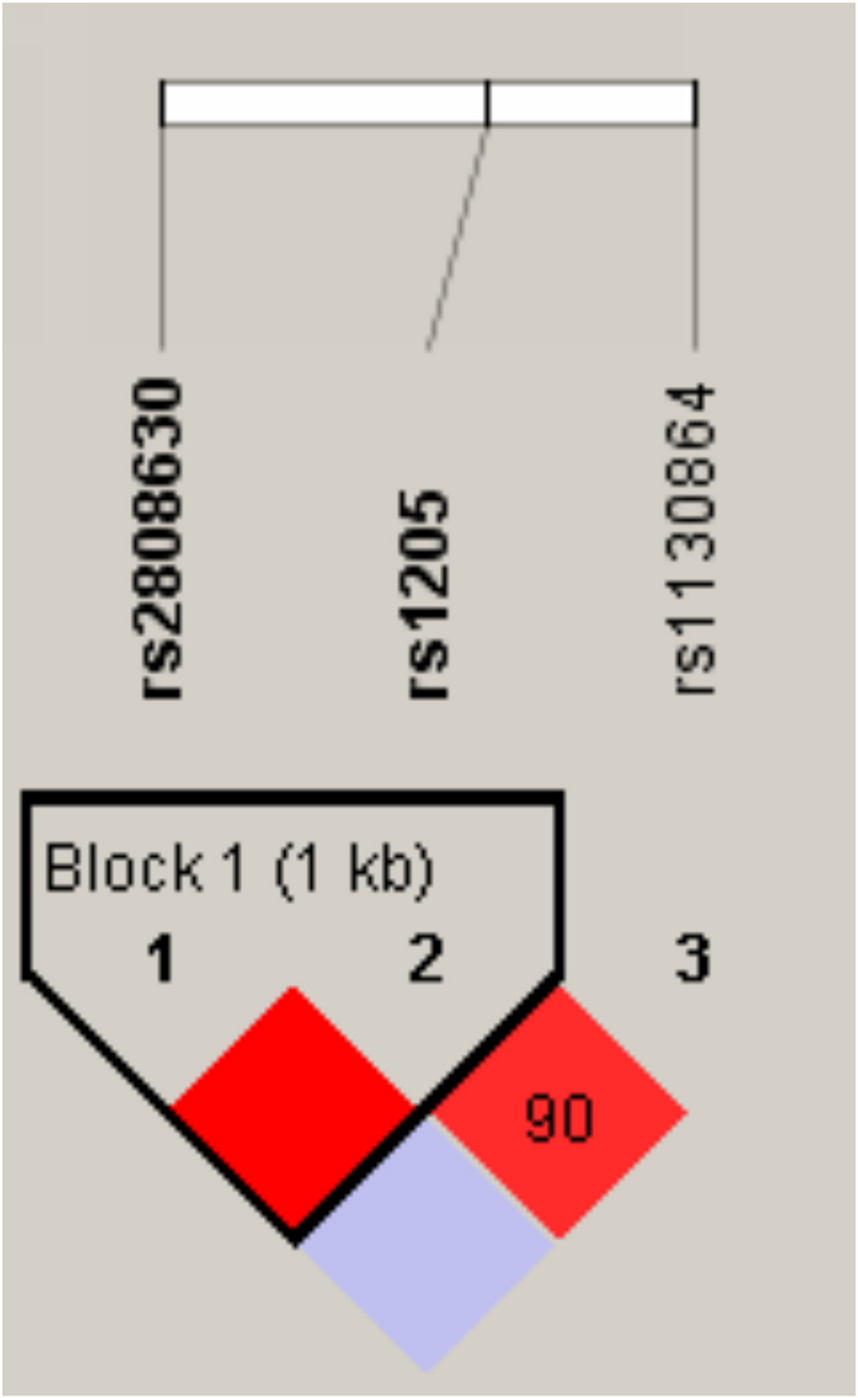
**Linkage disequilibrium of SNPs in the *CRP *gene using Haploview software.** The red color indicates the higher linkage disequilibrium (D’=1.0) between rs2808630 and rs1205.

**Figure 2:**
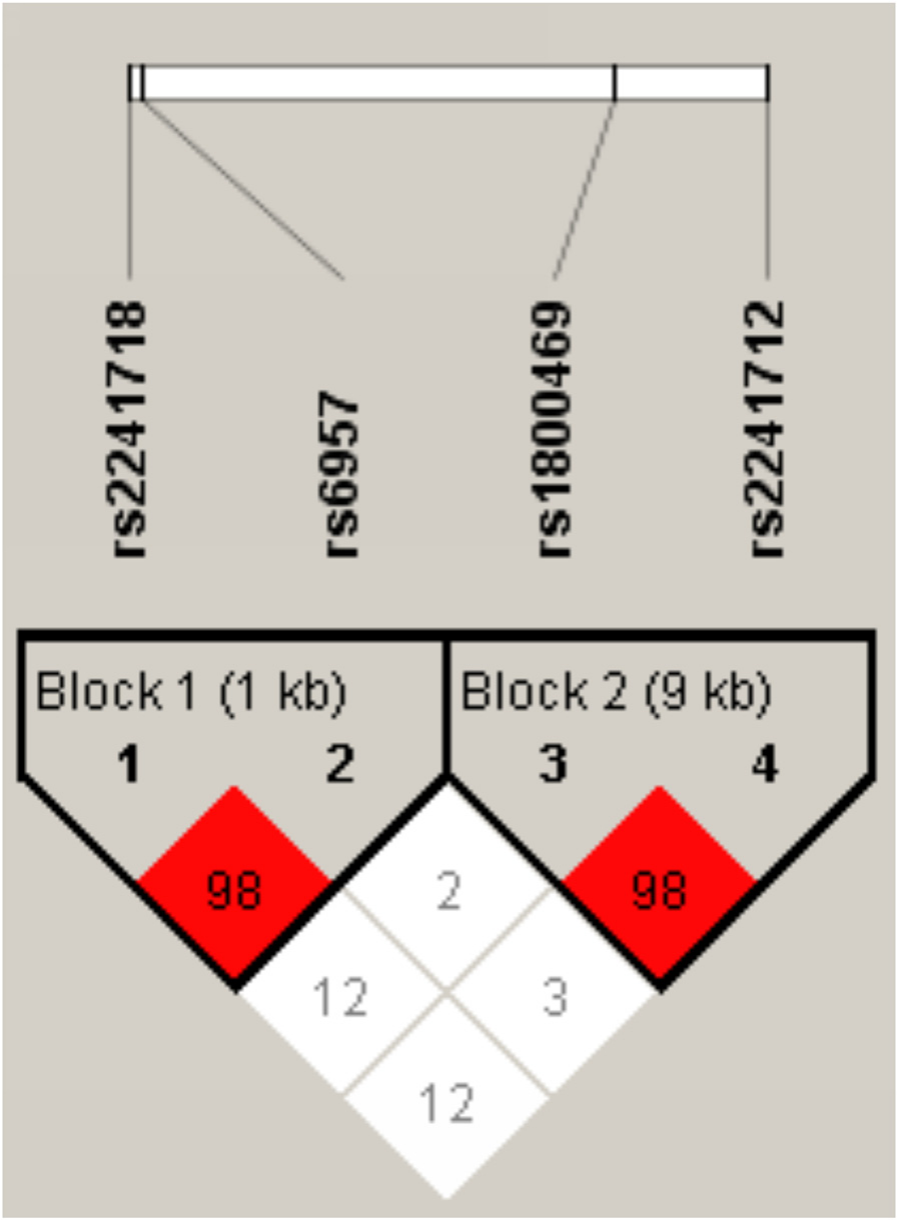
**Linkage disequilibrium of SNPs in the *TGF-β1 *and *CDC97 *genes using Haploview software.** The red color indicates the higher linkage disequilibrium (D’=0.98) between the rs2241718 and rs6957; between the rs1800469 and rs2241712.

Using Haploview software, *CRP* gene polymorphisms were determined to be in linkage disequilibrium (D’=1.0). Using PHASE software, haplotype frequencies for the polymorphisms rs1205 and rs2808630 of *CRP* at chromosome 1 were compared with respect to frequency between COPD patients and healthy controls. These two SNPs formed three haplotypes: CC, CT, and TT (Table 6 and Figure 1). Among them, only the TT haplotype was more frequently detected in controls (61%) compared with COPD patients (52%) (OR=0.69, 95% CI 0.49–0.98, p=0.0377). *TGF-β1* rs2241718 and *CDC97* rs6957 were in linkage disequilibrium (D’=0.98); they formed two haplotypes, GC and GT. These two haplotypes were respectively more frequent in healthy controls compared with COPD patients (OR=0.33, 95% CI 0.23–0.48, p=1.88×10^-9^) (Table 6 and Figure 2). *TGF-β1* rs1800469 and rs2241712 were also in linkage disequilibrium (D’=0.98); they formed TG and CA haplotypes. However, no significant differences between the two groups were detected (Table 6 and Figure 2).

**Table 6:** **Haplotypes of the *CRP *and *TGF-β1 *gene**

**SNPs(gene)**	**Haplotype**	**Control (n,%)**	**Case (n,%)**	**χ**^**2**^	**P value**	**OR**	**OR(95%CI)**
**rs2241718(TGF-β1)**	**GC**	107(50)	83(25)	36.0947	**1.88×10**^**-9**^	0.33	0.23-0.48
**rs6957(CDC97)**	Non-GC	106(50)	248(75)				
	**GT**	107(50)	83(25)	36.0947	**1.88×10**^**-9**^	0.33	0.23-0.48
	Non-GT	106(50)	248(75)				
**rs1800469(TGF-β1)**	**TG**	111(52)	159(48)	0.8615	0.3533	0.85	0.60-1.20
**rs2241712(TGF-β1)**	Non-TG	102(48)	172(52)				
	**CA**	94(44)	162(49)	1.2041	0.2725	1.21	0.86-1.71
	Non-CA	119(56)	169(51)				
**rs1205(CRP)**	**TT**	130(61)	172(52)	4.3163	**0.0377**	0.69	0.49-0.98
**rs2808630(CRP)**	Non-TT	83(39)	159(48)				
	**CC**	32(15)	60(18)	0.8883	0.3459	1.25	0.78-2.0
	Non-CC	181(85)	271(82)				
	**CT**	51(24)	96(29)	1.6822	0.1946	1.30	0.87-1.92
	Non-CT	162(76)	235(71)				

The p value with bold letters indicate those allele frequencies with significant differences between COPD and controls.

## Discussion

In this study, we sought to determine which of 76 SNPs we chose were associated with the development of COPD. Our case–control study verified that the following SNPs were associated with COPD: rs2353397 C, rs1800629 G, rs2241712 A, rs1205 C, rs20541 T, rs2070600 G, and rs10947233 G. The rs2353397 C allele was most strongly associated with COPD.

rs2353397 CC, CT genotypes of the *HHIP* gene were associated with susceptibility to COPD in the Chinese Han population. The *HHIP* gene is located at chromosome 4q31.21–31.3, position 145517578; it encodes the HHIP protein [17]. This protein is a critical regulator of the hedgehog (Hh) signalling pathway, which has been implicated in cell development, cell repair, and cancer development in multiple tissues [18]. The idea that COPD could be associated with inappropriate growth or structural defects in small airways makes *HHIP* an attractive candidate developmental gene. Several GWASs have also demonstrated that the 4q31 locus, which contains the *HHIP* gene, is associated with COPD and lung function [9-12]. A GWA meta-analysis for pulmonary function in 20,890 participants of European white ancestry revealed eight genes associated with COPD and concluded that *HHIP* is associated with FEV1/FVC [10]. Few studies prior to our current study have reported the SNPs of the *HHIP* gene related to COPD in an Asian population. Polymorphisms may lead to changes in gene expression, resulting in functional alteration and, subsequently, to COPD. According to Zhou et al. (2012) [19], significant decreases in expression of the *HHIP* gene at the mRNA and protein levels were observed in COPD lungs compared with lungs of smokers with normal lung function. The risk-associated haplotype confers decreased activity on the HHIP promoter, indicating that lower *HHIP* expression may exacerbate smoking-induced COPD pathogenesis. Lemjabbar-Alaoui et al. (2006) [20] demonstrated that hedgehog signaling proteins are critical mediators of cigarette smoke-induced disease, such as lung cancer and chronic airway inflammatory disease, and the expression levels of hedgehog signaling proteins are modulated by *HHIP*. Based on our current study of *HHIP* polymorphisms and the Hh signaling pathway, we need to further our mechanistic research in the context of smoking.

Our results also demonstrated that rs1800629 GG carriers of the *TNF-α* gene were at several times the risk for COPD compared with GA, AA carriers. *TNF-α* is critical in the regulation of inflammation; it induces a cascade of other inflammatory cytokines, chemokines, and other growth factors; it is important in the pathogenesis of many diseases. Several gene studies have also determined that the promoter polymorphism of *TNF-α* is associated with chronic bronchitis or the extent of emphysematous changes. Two of these studies were performed with Caucasian subjects, two with Japanese subjects [21-24]. The promoter polymorphism may have caused varied concentrations of serum TNF-α, which have been associated with induced sputum in bronchial biopsies and with bronchoalveolar lavage fluid in stable COPD patients and during exacerbations, compared with that of control subjects [25]. Some investigators have shown that *TNF-α* genotypes do influence the severity of infectious diseases, while others have concluded that polymorphisms of this gene promoter are of no functional consequence [26,27].

A polymorphism of the *TGF-β1* gene, rs2241712 A, tended to be a risk-associated allele in our study. TGF-β1 is one of the important cytokines involved in the inflammatory process of COPD. TGF-β1 expression is usually increased in the airways of patients. Su et al. (2005) [28] found that more carriers of the -800A allele, or fewer carriers of the -509T allele were detected among the COPD patients, but only 84 COPD cases and 97 healthy controls participated in their research. In addition, we used a Chinese Han population, while Su et al. recruited people in a general Chinese population. Van Diemen et al. (2010) [29] showed that the *TGF-β1* rs6957 SNP haplotype with the major allele of rs6957 and minor alleles of rs1800469 and rs1982073 were associated with COPD. The differences in study populations may explain these dissimilarities between our studies. Various studies have indicated that certain SNPs of the *TGF-β1* gene are functional and result in higher levels of circulating TGF-β1 [30,31].

The SNP rs20541 at *IL-13* gene exon 4 tended to be associated with COPD in our study. Genotype TT, CT carriers were at risk. IL-13 is a Th2 cytokine implicated in the recruitment of inflammatory cells from the blood to the lung, which may be involved in the pathogenesis of COPD. In experimental studies, the overexpression of IL-13 in the adult murine lung caused emphysema [32]. The number of IL-13+ cells was elevated in the bronchial submucosa of smokers with chronic bronchitis compared to asymptomatic smokers [33]. rs2066960, rs20541, and rs1295685 in the *IL-13* gene were associated with COPD risk and lower baseline lung function in the study by Beghé et al. (2010) [34], which used a Caucasian study population. We chose the same SNPs located in the *IL-13* gene, but our results showed that only rs20541 is of significance in susceptibility to COPD in the Chinese Han population. In addition, another study revealed the role of rs20541 in another chronic airway inflammatory disease (asthma), which indicates that the polymorphism in the coding region might contribute to airflow limitation [35].

The polymorphism of another inflammatory marker in the *CRP* gene, rs1205 C, was also a risk-associated allele according to our current study. Sunyer et al. (2008) [36] assessed the association between rs1205 and lung function; they demonstrated that the TT homozygous genotype in the *CRP* gene is associated with better lung function. Our result that the TT genotype protects people against COPD is similar to theirs because COPD is characterized by airflow limitation according to lung function. This polymorphism has been previously reported associated with varied levels of CRP in several studies [37]. Higher levels of CRP in peripheral blood may cause impaired lung function [38].

Two other SNPs, *AGER* rs2070600 and *PPT2* rs10947233, tended to be associated with COPD in our current study. rs2070600 GG and rs10947233 GG, GT carriers tended to develop COPD. An occidental GWAS demonstrated a role for the chromosome 6p21 locus including the *AGER* and *PPT2* genes in COPD development in smokers [39]. Repapi et al. (2010) [12] reported a meta-analysis of GWAS results from 20,288 participants and follow-up analyses in 54,276 participants; they identified five novel, genome-wide, significant loci for pulmonary function containing *AGER* rs2070600, but their analysis subject group did not include Asians. In addition, rs2070600 was associated with severe COPD in a study of Caucasian smokers from Poland [40]. The two SNPs of *AGER* and *PPT2* were also identified associated with FEV1/FVC in the 2009 work of Hancock et al. [10].

In addition, our study revealed some haplotypes composed of rs1205 and rs 2808630 in the *CRP* gene, rs2241718 and rs 6957 at the *TGF-β1* and *CDC97* genes. In our future research, we will further analyze our data with regard to lung function.

Our research had some limitations. First, a larger sample size would have improved quality of the results. Second, although we selected 97 SNPs for the study and found some loci related to the disease, further GWASs of COPD are needed in the Chinese Han population to identify more associated polymorphisms. It is likely that more genetic risk factors than those identified in this study contribute to the development of COPD. In our future work, we will further our investigation on gene function of the genetic factors related to the development of COPD.

## Conclusion

Our findings identified some genetic variants associated with COPD. This study has provided important information regarding the association of these polymorphisms to the susceptibility to COPD in the Chinese Han population. However, these findings need to be verified. These data emphasize the need for further research regarding gene function in COPD that will ultimately contribute to future gene therapies for this significant and costly disease in the Chinese Han population.

## Abbreviations

COPD: Chronic obstructive pulmonary disease; FEV1: Forced expiratory volume in 1s; FVC: Forced vital capacity; GWAS: Genome-wide association study; SNP: Single-nucleotide polymorphisms; MAF: Minor allele frequency; HHIP: Human hedgehog interacting protein.

## Competing interests

The authors declare that they have no competing interests.

## Authors’ contributions

YG provided blood samples, performed DNA extraction and the molecular genetic studies, and drafted the manuscript. YG provided blood samples, performed PCR, computational analysis. CMP carried out PCR and genotyping. YRQ provided blood samples. LR, QW, YC, TC, and LF provided blood samples. ZHJ performed DNA extraction. HYW, GCS, QJC participated in the design of the study and coordination of results, as well as editing the manuscript. All authors read and approved the final manuscript.

## Pre-publication history

The pre-publication history for this paper can be accessed here:

http://www.biomedcentral.com/1755-8794/5/64/prepub

## Supplementary Material

Additional file 1: Table S1 Primers of 97 single-nucleotide polymorphisms (SNPs) in multiplex PCR. Click here for file
