# Supplementary material for: Association of genetic polymorphisms with chronic obstructive pulmonary disease in the Chinese Han population: a case–control study
Source: BMC Med Genomics. 2012 Dec 26;5:64. doi: 10.1186/1755-8794-5-64 (PMC3582579; doi:10.1186/1755-8794-5-64)
Supplement: Additional file 1: Table S1 — Primers of 97 single-nucleotide polymorphisms (SNPs) in multiplex PCR. [file 1755-8794-5-64-S1.doc]

| **Supplemental Table 1: Primers of 97 single-nucleotide polymorphisms (SNPs) in multiplex PCR** | | | | |
| --- | --- | --- | --- | --- |
| ***SNP_ID*** | ***Gene*** | ***1st-Primer*** | ***2nd-Primer*** | ***UEP-Primer*** |
| rs1800610 | TNF-α | ACGTTGGATGGAAAGATGTGCGCTGATAGG | ACGTTGGATGCTTGCCACATCTCTTTCTGC | ATCCCCGTCTTTCTCCA |
| rs1799964 | TNF-α | ACGTTGGATGGGGAAGCAAAGGAGAAGCTG | ACGTTGGATGTACATGTGGCCATATCTCCC | tccaCTCCAGACCCTGACTTTTCCTTC |
| rs361525 | TNF-α | ACGTTGGATGATCAAGGATACCCCTCACAC | ACGTTGGATGCACACAAATCAGTCAGTGGC | gtgtCCCAGAAGACCCCCCTCGGAATC |
| rs1800629 | TNF-α | ACGTTGGATGGGTCCCCAAAAGAAATGGAG | ACGTTGGATGGATTTGTGTGTAGGACCCTG | GAGGCTGAACCCCGTCC |
| rs2808630 | CRP | ACGTTGGATGTCCCAGGGATGTAGGTTGAG | ACGTTGGATGTATTAAGGCCAGAGGCTGTC | GGCTGTCTACCAGACTA |
| rs1205 | CRP | ACGTTGGATGGTAGCCATCTTGTTTGCCAC | ACGTTGGATGTTTGTCAATCCCTTGGCTCC | AGTTTGGCTTCTGTCCTCA |
| rs1130864 | CRP | ACGTTGGATGATCTCCAAGATCTGTCCAAC | ACGTTGGATGTGGGAGCTCGTTAACTATGC | CTCGTTAACTATGCTGGGAAA |
| rs1059823 | SLC11A1 | ACGTTGGATGGCTGGTCTCAGGAACTGTC | ACGTTGGATGTGGCATGTATGACGTGACTG | tCGCGTGCAGGCAGCAGGAT |
| rs1130866 | SFTPB | ACGTTGGATGGCAGGAGGTGAGCTTGCAG | ACGTTGGATGAGTGCAACCAAGTGCTTGAC | TCGACTACTTCCAGAACCAGA |
| rs2353397 | HHIP | ACGTTGGATGGCTACCATAGCTGTTATATAC | ACGTTGGATGGGTACAGCTGCAAATAGCTC | aatcGCTCATTTCCACCATTATT |
| rs13147758 | HHIP | ACGTTGGATGCCTGTGATTGTATCAGTAAGA | ACGTTGGATGCTTGAGTTCTTGATGCTC | ttAGTTCTTGATGCTCAGTCCTA |
| rs2035901 | HHIP | ACGTTGGATGTGATTAAGGAAATAGAGTA | ACGTTGGATGTTTCCGCTTTGCACTTGGAG | cCCACGAGTTTGAAATCTGTAATAC |
| rs6537302 | HHIP | ACGTTGGATGGTGAAGTGCTTTGTAAGCTG | ACGTTGGATGGCATATTTTCCTTCTTCCCC | cccCCCCTCCTTTTTATGTTAGTATG |
| rs1032295 | HHIP | ACGTTGGATGTTTGCCCAGAGCTGCTAAAG | ACGTTGGATGTGGCAAAGAACATCTGGGAG | ttCAGTGTTTGGAGGAGAG |
| rs12504628 | HHIP | ACGTTGGATGCCCAGAAAATAAGAGTCTGC | ACGTTGGATGGGATTTGAGATTTAGAGTGCC | TTAGAGTGCCATTACCCCA |
| rs17019336 | HHIP | ACGTTGGATGCAGGTCTGCCATATCTTCAC | ACGTTGGATGTGAACAGTAGGAGAATCTTG | AGGAGAATCTTGAGTGGC |
| rs3749893 | TSPYL-4 | ACGTTGGATGCCTAAGTCCTTGAATTCACC | ACGTTGGATGGGTCACTCCTAGGATAATTG | cgAGGATAATTGAGAGAAAACATC |
| rs4987835 | Bcl-2 | ACGTTGGATGCTCTGGCATTATTTTCACAG | ACGTTGGATGTCTCCATTAGCAGGTGGTTG | cccaCTCTGCCCTGGCCCATGGCAT |
| rs2292566 | EPHX1 | ACGTTGGATGTGACATACATCCCTCTCTGG | ACGTTGGATGCAGGTGGAGATTCTCAACAG | acCAGATACCCTCACTTCAAGACTAA |
| rs1051740 | EPHX1 | ACGTTGGATGTGGCGTTTTGCAAACATACC | ACGTTGGATGTTGACTGGAAGAAGCAGGTG | gtatGTGGAGATTCTCAACAGA |
| rs868966 | EPHX1 | ACGTTGGATGTCCTGACCTATCCCAGCAAG | ACGTTGGATGTCAGTCACATAATCACCCTG | TGACAAATGTGAGCTCGAC |
| rs25882 | CSF2 | ACGTTGGATGTTAAAGGAAACTTCCTGTGC | ACGTTGGATGGCAGAAAGTCCTTCAGGTTC | tctaTTTGAAACTTTCAAAGGTGATA |
| rs829259 | PDE4D | ACGTTGGATGAGATTCTAGGCACTCTGTGG | ACGTTGGATGCCTCTTTCCAAAACATTCTC | ATTCCTCTCCTCCCTAC |
| rs6712954 | SERPINA2 | ACGTTGGATGGCTGTCCCCAGATCTTATTG | ACGTTGGATGGTGATTTCCACAGACCCTTG | CCCTTGAAATACACTGC |
| rs673400 | SERPINA2 | ACGTTGGATGTGATGGGTGCACACAGTATC | ACGTTGGATGGGTGTGAACCCACCATAAAG | TTGCCCACTGAAAAGGTCTA |
| rs7583463 | SERPINA2 | ACGTTGGATGCCTTATGAAAGCACATGGAG | ACGTTGGATGACTGAAAACACACGTGTGTC | TATGGATGAAAGTACAGTGAGG |
| rs2736100 | TERT | ACGTTGGATGACAAAGGAGGAAAAGCAGGG | ACGTTGGATGTGACACCCCCACAAGCTAAG | CCGTGTTGAGTGTTTCT |
| rs10069690 | TERT | ACGTTGGATGCTGTTTGAAACGGGTTCCTG | ACGTTGGATGTCATCTGAGGAGAGTGTGGG | GGTGAGGTGGACAGAGGT |
| rs34829399 | TERT | ACGTTGGATGTTGCTTAGTGTTACCCCCTG | ACGTTGGATGTTGTGCCATTACACTCCAGC | ggGAGACTGTCTCAATAAACAAAA |
| rs4246742 | TERT | ACGTTGGATGCCTTGATCCACTGTACATGC | ACGTTGGATGAGGCCACACAGCCATTTCTC | ggcaACACAGCCATTTCTCTCGAAG |
| rs2736118 | TERT | ACGTTGGATGTGCAGCTTCAGAGCCTCAG | ACGTTGGATGCTAGGTCCTCAGCACCAGT | ccCCAGTGCCACTCCTTACAGG |
| rs2736122 | TERT | ACGTTGGATGTCTCTCTCTGCCTCAAATC | ACGTTGGATGTTTCCGGAAAGGAGTCAAGC | ggCGCGTCAGGGAGATGCAAAC |
| rs2853677 | TERT | ACGTTGGATGCAATCCAGTCTGACAGTCGT | ACGTTGGATGGCAAGTGGAGAATCAGAGTG | ggGGAGAATCAGAGTGCACCAG |
| rs2853676 | TERT | ACGTTGGATGTTGGAGTGTCTCTGTCTGTC | ACGTTGGATGACTAAGACCCAAGAGGGAAG | cAGAGGGAAGTCTGACGAAGGC |
| rs1881457 | IL-13 | ACGTTGGATGTGCCTGGAGTGCCGCTACTT | ACGTTGGATGGGCCCTCTACTACAGATTAG | TTAGGAAACAGGCCCGTAG |
| rs1295685 | IL-13 | ACGTTGGATGCCTGAGTCTCTGAACCCTTG | ACGTTGGATGGAGTGTGTTTGTCACCGTTG | ggcTTGGGGAAGACTGTGGCTGCT |
| rs1800925 | IL-13 | ACGTTGGATGCAACACCCAACAGGCAAATG | ACGTTGGATGCATGTCGCCTTTTCCTGCTC | gggGCCTTTTCCTGCTCTTCCCTC |
| rs2066960 | IL-13 | ACGTTGGATGAACAATGCAGCCATTCTCCC | ACGTTGGATGAAGGAGCGGACTCTACTAAG | gaaTGTAAGGGCGGGCCTAT |
| rs20541 | IL-13 | ACGTTGGATGCCAGTTTGTAAAGGACCTGC | ACGTTGGATGGGTCCTGTCTCTGCAAATAA | ttCTTTCGAAGTTTCAGTTGAAC |
| rs16909898 | PTCH1 | ACGTTGGATGGAAGCAATCTGATGAACTCC | ACGTTGGATGCCAAGGTAATCTGCCACAAC | TCTGCCACAACTTAAGGCTC |
| rs10512249 | PTCH1 | ACGTTGGATGTCAGCCCAAAGAGAATGTGC | ACGTTGGATGGGTGTCCTTTTCTCGTTATG | TTATGCTTTGGTGGTTGA |
| rs35621 | ABCC1 | ACGTTGGATGAGAGAGAGAGGAAGGTGCTG | ACGTTGGATGTCCCAGTTTCCTCATTCCAC | cccaCTCATTCCACGTCCACT |
| rs2241718 | TGF-β1 | ACGTTGGATGACAATTTTCCCTTCCTCCGC | ACGTTGGATGCTCAGGACCCATGATAACAG | GACCCATGATAACAGCCTGTG |
| rs56155294 | TGF-β1 | ACGTTGGATGGAACAGGGCTGGTGTGGTG | ACGTTGGATGTCGAGGCCCTCCTACCTTTT | ccctGGGAGACCCCCAGCCCCT |
| rs1800469 | TGF-β1 | ACGTTGGATGTACAGGTGTCTGCCTCCTGA | ACGTTGGATGAAGAGGGTCTGTCAACATGG | gcGGGGGCAACAGGACACCTGA |
| rs2241712 | TGF-β1 | ACGTTGGATGAGCGCAAAAGACCCGCCTTC | ACGTTGGATGATAACGCATGCGCCTTATGG | AGGGAAGCGGGGTGGCTG |
| rs2277027 | ADAM19 | ACGTTGGATGTTGGCTTGGCCATATGTGTC | ACGTTGGATGGAAGAATTAAGGGCTGTGGC | ccccGATCAAAATGTGTCCTCAT |
| rs2280090 | ADAM33 | ACGTTGGATGCGTTCACCCCATGGAGTTG | ACGTTGGATGTTCTCCCTTCCCTCTCCAC | cccCCTCACTCACCCAGGGGCCAGG |
| rs2280091 | ADAM33 | ACGTTGGATGGTTGCTCAGCCCCAAAGATG | ACGTTGGATGAGGGCTGTCCAGTGGCTGT | cGCTGTGGGGCCCAACTCC |
| rs1435867 | PID1 | ACGTTGGATGACAGTGACTCATCAAAGCTC | ACGTTGGATGGAGTTGGGGAGAACAGATAG | ccccTGCTAAACTTGTATATTTCCTT |
| rs10498230 | PID1 | ACGTTGGATGCAAAAGGTCATTTACAAATT | ACGTTGGATGCCTGGAATATATAAGGGAGC | TACATGTAGATGTATGTCAAATT |
| rs3995090 | HTR4 | ACGTTGGATGAAACTCAGCCTCTTCTTCCC | ACGTTGGATGGGAAGTGGCCATATATATTC | atgCAACATAGACATATCATGGAGAT |
| rs6889822 | HTR4 | ACGTTGGATGTGGGAGATCAAGAGTGAAGG | ACGTTGGATGCCATTCACTCAAATAGCAGG | cTTGTTGAATTTTAGTGCACAA |
| rs1531697 | Bcl-2 | ACGTTGGATGACAAGAGAGACTAAAGGCCG | ACGTTGGATGCTGAATGAATAAGCCCGCTG | GAAGATGAGATTTCAAGGTC |
| rs1042713 | ARDB2 | ACGTTGGATGACACCTCGTCCCTTTCCTG | ACGTTGGATGGAACGGCAGCGCCTTCTTG | tgaAGCGCCTTCTTGCTGGCACCCAAT |
| rs3024791 | SFTPB | ACGTTGGATGTTAAGAGCCAGGCAGGAAGC | ACGTTGGATGTGGGTGTTCCCCTCCCATC | CCCAGGCCCCCTCTACT |
| rs511898 | ADAM33 | ACGTTGGATGTGCTGTATCTATAGCCCTCC | ACGTTGGATGAAAATACTGGGACTCGAGGC | CGAGGCCTGTGAATTCC |
| rs2853209 | ADAM33 | ACGTTGGATGTGTACTGGGAGGTAGAGGG | ACGTTGGATGCTTTGAACAGGAGGTTCCAG | GGCCTCCCAGTCAAGCG |
| rs6555465 | ADCY2 | ACGTTGGATGACCTTCACCTTAGAGACAGC | ACGTTGGATGCTGGGTGGCTTCCAATTTTC | TATCCATGTCATATCCAGC |
| rs10075508 | PDE4D | ACGTTGGATGGTTCCCCTCTGCAAGATATG | ACGTTGGATGACTCTTCCTTAATGGACAGC | CCTTAATGGACAGCCACATAAC |
| rs12899618 | THSD4 | ACGTTGGATGCCCAATACTCTGGCTGGAAT | ACGTTGGATGACAGTGTCCTGTGTGCTATG | ccTATTGCTTTTATGAGCCCTGAT |
| rs3091244 | SFXN1 | ACGTTGGATGTGAAGTAGGTGTTGGAGAGG | ACGTTGGATGCTTTGGCTATCTATCCTGCG | ATGGGAAATGGTAACATATTAAAC |
| rs8004738 | SERPINA1 | ACGTTGGATGCCTCCAACCTGGAATTCCTG | ACGTTGGATGTGAGCTGAACCAAGAAGGAG | gtTCGGGCCTCCGAGGAAGGCCTAGC |
| rs709932 | SERPINA1 | ACGTTGGATGCTGAGGAACAGGCCATTGC | ACGTTGGATGAGATCCATGAAGGCTTCCAG | gggtAAGGCTTCCAGGAACTCCTCC |
| rs4934 | SERPINA3 | ACGTTGGATGTTAGGGTGGCAGAGGACAG | ACGTTGGATGTCAGAGTTGAGAATGGAGAG | tcGAGAATGTTACCTCTCCTG |
| rs13706 | CDC6 | ACGTTGGATGGCTCAAGGTCATCCTGTTAC | ACGTTGGATGTCTGATTCCCAAGAGGGTTG | atgCACATATCCCAAGTCATCTCAGAA |
| rs7217852 | CDC6 | ACGTTGGATGGACACAGAAACTGCGTAGAG | ACGTTGGATGCAACGCACCAAGCAAGTCAT | gggaACCACCAATTGTGGTTCAG |
| rs2077464 | CDC6 | ACGTTGGATGGAAACAACCAAATTACCCCC | ACGTTGGATGTCCTTTAGGACAGTGATTGG | ATTCTTTCTTGTTTTGGAACATTT |
| rs2070600 | AGER | ACGTTGGATGACAGTGTGGCTCGTGTCCTT | ACGTTGGATGCACCGGAAAATCCCCTCATC | CGGAAGGAAGAGGGAGC |
| rs6957 | CDC97 | ACGTTGGATGCTATTTCCCAGAGGAGGAAG | ACGTTGGATGATCCTGCACTCTTGGCTTTC | gTCTTGGCTTTCTGAGAG |
| rs1042522 | P53 | ACGTTGGATGCAATGGTTCACTGAAGACCC | ACGTTGGATGTAGGAGCTGCTGGTGCAGG | TGCTGGTGCAGGGGCCACG |
| rs1695 | GSTP1 | ACGTTGGATGTGGTGGACATGGTGAATGAC | ACGTTGGATGGCAGATGCTCACATAGTTGG | GTTGGTGTAGATGAGGGAGA |
| rs4073 | IL-8 | ACGTTGGATGGTACTATATCTGTCACATGG | ACGTTGGATGCTGAAGCTCCACAATTTGGT | CACAATTTGGTGAATTATCAA |
| rs8192288 | SOD3 | ACGTTGGATGCTCACACCCCCATTTTACAG | ACGTTGGATGACTTCCTATCTGTGTACGCC | aagATCTGTGTACGCCTGAAGCAG |
| rs2571445 | TNS1 | ACGTTGGATGCAGCCATGCTGGGATTGATG | ACGTTGGATGAACAGTGGGCACCAACACTC | ccCTCCCCCTAGTCCTGGCTTCGGC |
| rs1003349 | MMP14 | ACGTTGGATGCTGCACCACAAAAAGGCAAC | ACGTTGGATGGACGTGGTTGTTTTAGCCTG | gggCTGAATCCAATTACAACCAAGAA |
| rs737693 | MMP12 | ACGTTGGATGCCTCTTGCAGAGCTTGTATG | ACGTTGGATGAGTTCTTTTCTGTCACAGGC | GCTGACTTTCTGGAGAAAT |
| rs2276109 | MMP12 | ACGTTGGATGGTCCGGGTTCTGTGAATATG | ACGTTGGATGTTGAGATAGATCAAGGGATG | gTAGATCAAGGGATGATATCAACT |
| rs1052443 | NT5DC1 | ACGTTGGATGGCCTATAGAGAAGTTGGGTC | ACGTTGGATGGTCTGTGAACAGGTACATGG | aaAGGTACATGGTACAACAAACTGAT |
| rs10947233 | PPT2 | ACGTTGGATGAGAGGTTGGCAAACTGTGAC | ACGTTGGATGAATGAATGTGCCTGTGTTCC | AGTAGCAGGCTGGATTT |
| rs1051730 | CHRNA3 | ACGTTGGATGCAGCAGTTGTACTTGATGTC | ACGTTGGATGCTCAAGGACTATTGGGAGAG | ATCATCAAAGCCCCAGGCTA |
| rs11106030 | DCN | ACGTTGGATGACTTGGACAGCTGCTCTTTG | ACGTTGGATGAATGTGGGCTGTCAATACTG | CTGTCAATACTGATAATTCCTTT |
| rs584367 | sPLA2s | ACGTTGGATGATGACTGCTGCTATGACCAC | ACGTTGGATGCCCCTGGGAAAAGTTGTATC | TCTGTAATAGTCCTTGTAGATGC |
| rs9904270 | CDC6 | ACGTTGGATGTCTGCAGACTATGCCTTCCC | ACGTTGGATGCATGGCCTTCCCCTTAGAG | ccTGGCCTTCCCCTTAGAGAAGAGC |
| rs2395730 | DAAM2 | ACGTTGGATGATACAGAGGTAAAGTGTGGG | ACGTTGGATGACCCCCAGTTTTGAGAGCTG | caCCCAGTTTTGAGAGCTGCTGGTCT |
| rs3817928 | GPR126 | ACGTTGGATGGGTGCCAAGGTAAAGAGATG | ACGTTGGATGTGCAGCGTGTAATGTCTCAG | ttATGTTTCACTTGTGAGGGACTGAA |
| rs11155242 | GRP126 | ACGTTGGATGGGCTACTTTCTATCCATTTC | ACGTTGGATGAGCTGTTCAAAGCTTTCTGC | TAATGCATTATTCAACAAACATT |
| rs7776375 | GPR126 | ACGTTGGATGACTTTGCACCGTGTGGTAAC | ACGTTGGATGGTCAAATTAATGATCCCCCC | attcATGATCCCCCCCAACAC |
| rs6937121 | GPR126 | ACGTTGGATGCAGTAAAATTCTGATAGCC | ACGTTGGATGGAAAGATTGGCACAACTGTC | gCAAATCTATTATCTAATTTGTCCA |
| rs1042714 | ARDB2 | ACGTTGGATGCTTCTTGCTGGCACCCAATG | ACGTTGGATGAGACATGACGATGCCCATGC | CCACACCTCGTCCCTTT |
| rs1800796 | IL-6 | ACGTTGGATGTCTTCTGTGTTCTGGCTCTC | ACGTTGGATGACGCCTTGAAGTAACTGCAC | GCAGTTCTACAACAGCC |
| rs2236307 | MMP14 | ACGTTGGATGAATGCCCCTCGTGTTTTCTG | ACGTTGGATGTTATCAGGAACAGAAGGCCG | GTGGGGAACCCTGACTC |
| rs2236302 | MMP14 | ACGTTGGATGCTCGAGCATTCCAGTGACC | ACGTTGGATGTCGGGCAGCACAAAATTCTC | gcggGTCCATCCACTGGTAAAA |
| rs2230054 | IL-8RB | ACGTTGGATGTCCTCATGAGGGTGTCTGC | ACGTTGGATGTCATCTTTGCTGTCGTCCTC | TCGTCCTCATCTTCCTGCT |
| rs1422795 | ADAM19 | ACGTTGGATGGCGTTGTCATGGTACTTCTG | ACGTTGGATGCCATATTCTACCCTCTGGTC | gCTACCCTCTGGTCCTTTCTC |
| rs6830970 | FAM13A | ACGTTGGATGATCTGCCATGCTAAGTGGAC | ACGTTGGATGCCTTCTACTCCTCCACAATG | GAAGCTGTCCAGTAAACGAAA |
| rs2869967 | FAM13A | ACGTTGGATGAAGAGCAGAGCTCTTGGAAC | ACGTTGGATGTGAGCCCCCATTTTCTAACC | tacCCCAGGATCCTGAATACATCCCTT |

1. Van Krugten M, Pennings HJ, Huizinga TW, et al.: **Tumor necrosis factor-alpha +489G/A gene polymorphism is associated with chronic obstructive pulmonary disease.** Respir Res 2002, **3:**29.
2. Wood AM, Simmonds MJ, Bayley DL, et al.: **The TNF-alpha gene relates to clinical phenotype in alpha-1-antitrypsin deficiency.** Respir Res 2008, **9:**52.
3. Sakao S, Tatsumi K, Igari H, Watanabe R, Shino Y, Shirasawa H, Kuriyama T: **FCCP Association of tumor necrosis factor gene promoter polymorphism with low attenuation areas on high-resolution CT in patients with COPD.** Chest 2002, **122(2):**416–420.
4. Yanbaeva DG, Dentener MA, Spruit MA, et al.: **IL-6 and CRP haplotypes are associated with COPD risk and systemic inflammation: a case-control study.** BMC Med Genet 2009, **10:**23.
5. Sunyer J, Pistelli R, Plana E, Andreani M, Baldari F, Kolz M, Koenig W, Pekkanen J, Peters A, Forastiere F: **Systemic inflammation, genetic susceptibility and lung function.** Eur Respir J 2008, **32(1):**92–97.
6. Kim EJ, Kim KM, Park SH, et al.: **SLC11A1 polymorphism are associated with the risk of chronic obstructive pulmonary disease in a Korean population.** Biochem Genet 2008, **46:** 506–-519.
7. Hersh CP, Demep DL, Lange C, et al.: Attempted replication of reported **chronic obstructive pulmonary disease candidate gene associations.** Am J Respir Cell Mol Biol 2005, **33:**71-78.
8. Hancock DB, Eijgelsheim M, Wilk JB, Gharib SA, Loehr LR, Marciante KD, Franceschini N, van Durme YMTA, Chen TH, Barr RG, Schabath MB, Couper DJ, Brusselle GG, Psaty BM, van Duijn CM, Rotter JI, Uitterlinden AG, Hofman A, Punjabi NM, Rivadeneira F, Morrison AC, Enright PL, North KE, Heckbert SR, Lumley T, Stricker BHC, O’Connor GT, London SJ: **Meta-analyses of genome-wide association studies identify multiple loci associated with pulmonary function.** Nature Genetics 2010, **42(1):**45–52.
9. Sata M, Takabatake N, Inoue S, et al.: **Intronic single-nucleotide polymorphism in Bcl-2 are associated with chronic obstructive pulmonary disease severity.** Respirology 2007, 12:34–41.
10. Vibhuti A, Arif E, Deepak D, et al. **Genetic polymorphisms of GSTP1 and mEPHX correlate with oxidative stress markers and lung function in COPD**. Biochem Biophys Res Comm 2007, **359:**136–142.
11. Brogger J, Steen VM, Eiken HG, et al.: **Genetic association between COPD and polymorphisms in TNF, ADRB2 and EPHX1.** Eur Respir J 2006, **27:**682–-688.
12. Shen M, Vermeulen R, Chapman RS, et al.: **A report of cytokine polymorphisms and COPD risk in Xuan Wei, China.** Int J Hyg Environ Health 2008, **211:**352–356.
13. Homma S, Sakamoto T, Hegab AE, et al.: **Association of phosphodiesterase 4D gene polymorphisms with chronic obstructive pulmonary disease: relationship to inerleukin 13 gene polymorphism.** Int J Mol Med 2006, **18:**933–939.
14. DeMeo D, Mariani T, Lange C, et al.: **The SERPINE2 gene is associated with chronic obstructive pulmonary disease.** Proc Am Thorac Soc 2006,3:502.
15. Zhu G, Warren L, Aponte J, et al.: **The SERPINE2 gene is associated with chronic obstructive pulmonary disease in two large populations.** Am J Respir Crit Care Med 2007, 176:167–173.
16. Beghé B, Hall IP, Parker SG, Moffatt MF, Wardlaw A, Connolly MJ, Fabbri LM, Ruse C, Sayers I: **Polymorphisms in IL13 pathway genes in asthma and chronic obstructive pulmonary disease.** Allergy 2010, **65(4):**474–481.
17. Siedlinski M, Boezen HM, Boer JM, et al. **ABCC1 polymorphisms contribute to level and decline of lung function in two population-based cohorts.** Pharmacogenet Genomics 2009, 19:665–684.
18. Su ZG, Wen FQ, Feng YL, Xiao M, Wu XL: **Transforming growth factor-beta1 gene polymorphisms associated with chronic obstructive pulmonary disease in Chinese population.** Acta pharmacol sin 2005, **26(6):**714–720.
19. Van Diemen CC, Postma DS, Aulchenko YS, Snijders PJLM, Oostra BA, van Duijin CM, Boezen M: **Novel strategy to identify genetic risk factors for COPD severity: a genetic isolate.** Eur Respir J 2010, **35(4):** 768–775.
20. Repapi E, Sayers I, Wain LV, Burton PR, Johnson T, Obeidat M et al.: **Genome-wide association study indentifies five loci associated with lung function.** Nature Genetics 2010, **42(1):**36–44.
21. Vacca G, Schwabe K, Duck R, et al.: **Polymorphisms of the beta2 adrenoreceptor gene in chronic obstructive pulmonary disease.** Ther Adv Respir Dis 2009, **3:**3–10.
22. Guo X, Lin HM, Lin Z, et al. **Surfactant protein gene A, B, and D marker alleles in chronic obstructive pulmonary disease of a Mexican population.** Eur Respir J 2001, **18:** 482–490.
23. Sadeghnejad A, Ohar JA, Zheng SL, et al.: **Adam33 polymorphisms are associated with COPD and lung function in long-term tobacco smokers.** Respir Res 2009, **10:**21.
24. Chappell S, Daly L, Morgan K, et al.: **Cryptic haplotypes of SERPINA1 confer susceptibility to chronic obstructive pulmonary disease.** Hum Mutat 2006, **27:**103–109.
25. Ishii M, Teramoto M, Hosoi F: **Association between alpha-1-antichymotrypsin polymorphism and susceptibility to chronic obstructive pulmonary disease.** European Journal of Clinical Investigation 2000, **30:**543–548.
26. Takabatake N, Toriyama S, Igarashi A, et al.: **A novel polymorphism in CDC6 is associated with the decline in lung function of ex-smokers in COPD.** Biochem Biophys Res Commun 2009, **381:**554–559.
27. Van Diemen CC, Postma DS, Vonk JM, et al:. **Decorin and TGF-beta1 polymorphisms and development of COPD in a general population.** Respir Res 2006, **7**: 89.
28. Lee YL, Chen W, Tsai WK, et al. **Polymorphisms of p53 and p21 genes in chronic obstructive pulmonary disease.** J Lab Clin Med 2006, **147:**228–233.
29. Lakhdar R, Denden S, Knani J, et al.: **Relationship between glutathione S-transferase P1 polymorphisms and chronic obstructive pulmonary disease in a Tunisian population.** Genet Molec Res 2010, **9:**897–907.
30. Dahl M, Bowler RP, Juul K, et al.: Superoxide dismutases 3 polymorphism associated with reduced lung function in two large population. Am J Respir Crit Care Med 2008, **178:**906–912.
31. Saitoh W, Sakamoto T, Hegab AE, et al.: **MMP14 gene polymorphisms in chronic obstructive pulmonary disease.** Int J Mol Med 2006, **17:**621–626.
32. Hunninghake G, Cho M, Tesfaigzi Y, et al.: **A functional MMP12 polymorphism is associated with lung function throughout life and delayed onset of COPD.** American Journal of Respiratory and Critical Care Medicine 2009,179:A3004.
33. Pillai SG, Ge DL, Zhu GH, Kong XY, Shianna KV, Need AC, Feng S, Hersh CP, Bakke P, Gulsvik A, Ruppert A, Lødrup Carlsen KC, Roses A, Anderson W, ICGN Investigators, Rennard SI, Lomas DA, Silverman EK, Goldstein DB: **A genome-wide association study in chronic obstructive pulmonary disease (COPD): identification of two major susceptibility loci.** PLoS Genet 2009, **5(3):**e1000421.
34. Takabatake N, Sata M, Inoue S, et al.: **A novel polymorphism in secretory phospholipase A2-IID is associated with body weight loss in chronic obstructive pulmonary disease.** Am J Respir Crit Care Med 2005, **172:**1097–1104.
35. Matheson MC, Ellis JA, Raven J, et al.: **Beta2-adrenergic receptor polymorphisms are associated with asthma and COPD in adults.** J Hum Genet 2006, **51:**943–951.
36. Yanbaeva DG, Dentener MA, Spruit MA, et al.: **IL6 and CRP haplotypes are associated with COPD risk and systemic inflammation: a case-control study.** BMC Med Genet 2009, **10:**23.
37. Stemmler S, Arinir U, Klein W, et al.: **Association of interleukin-8 receptor alpha polymorphisms with chronic obstructive pulmonary disease and asthma.** Genes Immun 2005, **6:**225–230.
